# Exploring sex differences: insights into gene expression, neuroanatomy, neurochemistry, cognition, and pathology

**DOI:** 10.3389/fnins.2024.1340108

**Published:** 2024-02-21

**Authors:** Muataz S. Lafta, Jessica Mwinyi, Oreste Affatato, Gull Rukh, Junhua Dang, Gerhard Andersson, Helgi B. Schiöth

**Affiliations:** ^1^Department of Surgical Sciences, Functional Pharmacology and Neuroscience, Uppsala University, Uppsala, Sweden; ^2^Centre for Women’s Mental Health, Uppsala University, Uppsala, Sweden; ^3^Department of Behavioural Sciences and Learning, Linköping University, Linköping, Sweden; ^4^Department of Clinical Neuroscience, Karolinska Institute, Stockholm, Sweden

**Keywords:** sex differences, genetics, neuroanatomy, neurochemistry, cognition, pathology

## Abstract

Increased knowledge about sex differences is important for development of individualized treatments against many diseases as well as understanding behavioral and pathological differences. This review summarizes sex chromosome effects on gene expression, epigenetics, and hormones in relation to the brain. We explore neuroanatomy, neurochemistry, cognition, and brain pathology aiming to explain the current state of the art. While some domains exhibit strong differences, others reveal subtle differences whose overall significance warrants clarification. We hope that the current review increases awareness and serves as a basis for the planning of future studies that consider both sexes equally regarding similarities and differences.

## Introduction

The study of sex differences is one of the most challenging and debated topics in neuroscience. The importance of research on sex differences in brain and behavior has often been underestimated over the past decades, but in recent years our knowledge about the influence of sex on brain structure, function and chemistry has grown tremendously. We have witnessed an increasing number of findings about sex differences and their importance for the risk and course of human diseases (Heidari et al., 2017; Clayton, 2018). Advances in analytic techniques, and a wider access to their use, have granted the opportunity to study the brain in more detail and to evaluate more precisely differences between men and women. However, despite decades of research, sex differences in brain function are only partly understood.

New methodological approaches, from gene modification in mice to voxel-based morphometry analyses of human imaging data, have revealed previously undetected sex differences (Shah et al., 2004; Bielsky et al., 2005). Sex differences exist also in a wide variety of behavioral traits and in the incidence and prevalence of many diseases. The probabilities of developing certain mental disorders differ substantially between men and women and many of the diagnoses show sex-related differences which comprise differences in the age of onset, diagnostic criteria, clinical presentation, disease progression, severity and treatment efficacy (Klein and Corwin, 2002; Shors, 2002; Bale and Epperson, 2015). For these reasons, the neurobiological study of sex differences is of critical importance to understand sex-specific pathways and mechanisms underlying sex-specific differences in the type and prevalence of mental disorders. Furthermore, many drugs on the market have been developed using a one-size-fits-all approach, in earlier times often based on trials including only males within certain age ranges, which has resulted in an increased adverse event risk and reduced therapy efficacy in females, pointing to the necessity to better understand biologically caused differences between sexes (Tharpe, 2011; Yu et al., 2016). In recent years, the number of studies investigating sex differences in general has increased. However, more studies are needed to be able to individually adjust medical treatment options nowadays available. The aim of this narrative review is to shed light on important sex differences in the human brain, discussing findings on the genetic, cognitive and behavioral level, and their influence on sex-dependent health, education, and lifestyle.

### Sexual differentiation of the brain – genetics and hormones

Sex differences in brain structure are observed early in the life course (Knickmeyer et al., 2014). Developmental sex differentiation of the brain is suggested to be of multifactorial nature, including the influence of genes on sex chromosomes or sex hormones impacting the developing and adult brain. One of the main mechanisms leading to sexual differentiation is induced by the genetic sex (XX and XY) that triggers the differentiation of the gonads, which in turn secrete gonadal hormones that induce sex-specific functional differences of different tissues. However, this mechanism explains only in part sex-specific differences induced in early development. A more accurate model of sexual differentiation needs to include also inherent differences in the genome and the impact of the environment on sex-specific biology (McCarthy and Arnold, 2011; Lombardo et al., 2012).

### Sex chromosomes as a main player in establishing sex differences

Sex chromosomes are thought to be the primary source of variance in the brain between sexes (Arnold et al., 2003; Cisternas et al., 2018). Much attention has been paid in identifying sex chromosomes as a major player in controlling molecular pathways responsible for the expression of sex differences (Dewing et al., 2003; Glickman et al., 2005; Scholz et al., 2006). Besides its influence on gonadal differentiation and steroid hormone production, chromosome complement appears to be a relevant variable that contributes to sex differences in brain and behavior (Arnold et al., 2016)_._ From a biological perspective, sex is defined through the size of gametes within a species. Animals with larger gametes (i.e., eggs) are female and those with smaller gametes (i.e., sperm) are male (Smith, 1978). In mammals, eggs are formed in ovaries and sperm in testes. Thus, the gonad type is often used as a shorthand for defining sex. Which gonad develops is determined by the chromosomal sex (XX or XY). If a Y chromosome is present, a gene cascade is initiated and causes gonads to become testes. In the absence of a Y chromosome, another cascade leads to the differentiation of ovaries (Brennan and Capel, 2004). An important gene is the gene *SRY* on the Y chromosome. Abundance and expression of this evolutionarily conserved gene drives sexual differentiation of the gonads (ovaries in females and testes in males) and the expression of sex steroid hormones. The testes produce the androgenic steroid hormone testosterone, which is responsible for masculinization of the external genitalia, internal duct systems, and other male specific somatic characteristics (Jost, 1978).

In the brain, *SRY* drives the production of catecholamines by dopaminergic neurons of the substantia nigra as shown in mouse models and in models using the NT2 human teratocarcinoma cell line. As only males carry the *SRY* gene, it is hypothesized that the gene may contribute to the higher susceptibility of males to dopamine disorders such as Parkinson disease and schizophrenia (Czech et al., 2012). The influence of sex chromosomes on somatic genes can partly be explained through differences in the dosage of X chromosome genes in males and females. Despite a 2:1 ratio of X chromosome genes in females and males, X chromosome genes are normally equally expressed in mammals through a compensatory system in gene expression (Nguyen and Disteche, 2006). The X chromosome bound gene *Xist* induces inactivation of one of the X chromosomes in females by producing non-coding RNA that initiates a cascade of heterochromatinizing, leading to a silent inactive X chromosome (Chang et al., 2006). *Xist* is rarely included on lists of genes that cause sexual differentiation, even though its role in causing mosaicism in females and not in males has been emphasized (Migeon, 2007). However, some of the X chromosome genes do escape from the described compensation system of inactivation leading to increased gene expression levels in females relative to males (Tukiainen et al., 2017). Since there is accumulating evidence on X chromosome genes being involved in mental function and the control of sex differences during brain development (Skuse, 2005), the escape of inactivation can contribute to sex differences in brain function, cognition and pathology (Leitão et al., 2022). In male mammals, on the other hand, there are genes located on the non-recombining region of the Y chromosome (NRY) that do not exist in females. These genes could potentially influence the development of masculine neural patterns in the brain (Xu et al., 2002).

### The role of gene expression, differential splicing, and epigenetic control in establishing sex differences

Several species show sex-dependent differences in the expression of genes in various tissues, including the brain (Isensee and Ruiz, 2007; Santos et al., 2007; Reinius et al., 2008). Sex-biased expression has been observed across various developmental stages, such as prenatal, early childhood, puberty, and adulthood, with a substantial number of genes (>2,000 genes) exhibiting between-sex expression divergence at all developmental stages, with the highest number (4,164 genes) observed during puberty (Shi et al., 2016). It has also been observed in the context of diseases, including depression, schizophrenia, and at least 17 types of cancer (Ma et al., 2016; Qin et al., 2016; Labonté et al., 2017), as well as under different environmental conditions, such as the response of hepatocytes to hepatotoxicants (Mennecozzi et al., 2015). Additionally, age influences gene expression differently in male and female brains, with males exhibiting more global gene changes. Gene ontology analysis reveals that the male brain is characterized by the down-regulation of genes heavily enriched in energy production and protein synthesis/transport categories, while both sexes show increased immune activation, notably higher in females (Berchtold et al., 2008). According to more recent genome-wide expression studies, an extensive sex difference in gene expression levels was discovered in the rodent brain, which preceded the gonadal differentiation (Dewing et al., 2003), indicating that the differences in expression were independent of hormone action.

Differential splicing and epigenetic control are other proposed molecular mechanisms for regulating sex-biased expression differences. Alternative splicing allows the production of a variety of different proteins from one gene only. Interindividual differences in RNA splicing play an important role in the development of complex traits such as autoimmune diseases, including Crohn disease, type 1 diabetes and rheumatoid arthritis (Gamazon and Stranger, 2014; Manning and Cooper, 2017). Sex differences in splicing have been reported in skeletal muscles (Lindholm et al., 2014), liver (Blekhman et al., 2010) and human brain (Trabzuni et al., 2013) and may contribute to sex-differentiated phenotypes. In the adult human brain, sex differences in gene expression and splicing were widespread, detectable in all major brain regions, and involving 2.5% of all expressed genes (Trabzuni et al., 2013).

Another important mechanism contributing to sex differences is the inherited epigenetic regulation of gene expression. Epigenetic control determines the expression of genes and is a mechanism by which the genome can respond to environmental stimuli. In other words, individuals (or cells) with the same genes may develop very different phenotypes based on environmental interventions at key stages of development (Jaenisch and Bird, 2003). Epigenetic modifications play an important role in the establishment and maintenance of sex differences in brain and body and include mechanisms such as DNA methylation (Tapp et al., 2013; Hall et al., 2014), histone modifications, nucleosome repositioning, chromatin accessibility, mechanisms involving non-coding RNA, and RNA and DNA editing (Vigé et al., 2008). It has been shown that epigenetic marks are associated with the development of several mental disorders in a sexually differentiated manner, including Angelman syndrome, Prader-Willi syndrome, autism, and bipolar disorder (Davies et al., 2001).

### Hormonal regulation of sex differences

Sex hormone expression and synthesis is one of the key drivers of sexual differentiation at the molecular and phenotypic level. Males have higher circulating levels of the main male hormone testosterone derived from the testis. Testosterone is also produced by the ovaries of females (Burger, 2002). The brain also has the capacity to locally generate the androgen dihydrotestosterone independently of the gonads, from yet unknown precursors (Okamoto et al., 2012). On the other hand, estrogens, predominantly found in women, are produced in a cyclic pattern from the female ovary but also in several extragonadal sites, including mesenchymal cells within adipose tissue including that of the breast, bone’s osteoblasts and chondrocytes, the vascular endothelium and aortic smooth muscle cells, and various locations within the brain (Simpson, 2003). Sex hormones are generally thought to shape the brain in three major ways. First, by organizational effects, such as defining brain structures during neurodevelopment (Wallen, 2005). Later, by altering intrinsic functions in the brain, such as modulating of hippocampal spines by the cyclic pattern of estradiol levels (Woolley, 1998). And lastly, by the interaction of these sexually determined traits with the social environment at different sensitive periods, while also considering neural and psychological mediators, and including the influence of gendered socialization (Berenbaum and Beltz, 2016).

Different mechanisms of sex hormone action in the brain have been proposed to influence neurogenesis, cell migration, synaptogenesis, axon guidance, cell death and cell differentiation (Flerkó, 1971; McEwen, 1972). Moreover, both estrogens and androgens have neuroprotective effects (Pike et al., 2008). Steroid hormones signaling is primarily achieved by binding to nuclear receptors, followed by dimerization and docking at hormone response elements on gene promoters to finally regulate gene transcription (Stanisić et al., 2010; Grimm et al., 2016). Estrogens exert their function by binding to the estrogen receptors alpha and beta. The receptors bind to specific DNA sequences located in the regulatory regions of estrogen-responsive genes (estrogen response elements (ERE)) resulting in the transcriptional modulation of the target genes (Bourdeau et al., 2004). Similarly, androgens, including testosterone, exert their effects by binding to androgen receptors. The receptors bind to different sets of androgen responsive elements (AREs) influencing the expression of target genes (Stanisić et al., 2010). As shown for mice and rats, treating new-born females with testosterone partially masculinizes the DNA methylation pattern present in adulthood (Schwarz et al., 2010; Ghahramani et al., 2014). In fact, steroid hormones alter the expression or activity of enzymes involved in epigenetic modifications, which may explain how sex hormones affect the epigenome. Specifically, research has found that gonadal steroids primarily reduce the activity of DNA methyltransferase (Dnmt) enzymes, leading to decreased DNA methylation and the release of masculinizing genes from epigenetic repression (Nugent et al., 2015).

Estrogen plays a critical role in the sexual differentiation of the brain (McEwen, 2001) and thus likely contributes to sex differences in brain morphology and neurochemistry. Progress to understand the steroid-mediated brain differentiation has been made by using genetically modified mice to elucidate cellular mechanisms and downstream effects, and to characterize the behavioral and neuronal effects of steroid hormones. For instance, loss of the estrogen receptor alpha (*Esr1*) greatly reduces sex behavior in male mice (Ogawa et al., 1998). While loss of estrogen receptor beta (*Esr2*) alone has no effect on male reproduction-related behavior. However, dysfunction of both estrogen receptors results in complete loss of male sex behavior (Ogawa et al., 2000) as Esr2 is specifically involved in the suppression of female sex behavior (defeminization) in male mice (Kudwa et al., 2005).

While the brain was for many years not regarded as a target for estrogens and other hormones, except the hypothalamus, we now know that the entire brain is a target for gonadal hormones (McEwen, 2007). Sex hormones act throughout the entire brain of both males and females (McEwen and Milner, 2017). There is a main impact of sex hormones on brain development and plasticity (Marino et al., 2006). The trophic effects of ovarian hormones emerge early in brain development and remain throughout adolescence (Juraska et al., 2013) and adulthood (Wise et al., 2008). Many of these actions occur in brain regions involved in motivation (Sakaki and Mather, 2012), cognition (Berman et al., 1997), learning (Hu et al., 2007) and memory (Liu et al., 2008), emotion (Amin et al., 2006), and motor control (Horstink et al., 2003). Estrogens and progesterones exert trophic effects on brain development throughout adolescence and adulthood and they act together to enhance neuronal function through mechanisms such as synapse formation and reduction, enhancing synaptic transmission and exerting neuroprotective effects (Singh and Su, 2013; Toffoletto et al., 2014; Rossetti et al., 2016). Progesterone receptors have also been identified in brain regions relevant for cognition, including the frontal cortex, hypothalamus, thalamus, hippocampus, amygdala, and cerebellum (Brinton et al., 2008). Animal studies assessing the effects of progesterone administration to ovariectomized rats are not as established compared to studies with estradiol. However, results generally suggest that progestins and progesterones can have beneficial effects on spatial cognitive performance that may be dependent on the timing of administration and the type of progestin/progesterone (Toffoletto et al., 2014).

Animal studies have shown that hormonal changes in puberty exert profound effects on brain maturation and behavior (Sisk and Foster, 2004). The hormonal events of puberty trigger a second period of structural reorganization in the human brain (Spear, 2000). Life stages characterized by changes in hormone levels, such as puberty, pregnancy, postpartum, and menopause, are critical periods known to exacerbate or trigger the new onset of certain pathologies, including asthma (Murphy and Gibson, 2008), obsessive-compulsive disorder (Guglielmi et al., 2014) and depression (Schiller et al., 2015). For example, the incidence of asthma peaks early in boys, before puberty, after which the incidence in women is double as high compared to men (Zein and Erzurum, 2015). Menopause, a stage of life marked by changes in female hormone levels, has been linked with changes in the expression of several immune and metabolism genes in adipose (Gomez-Santos et al., 2011) and bone (Kósa et al., 2009) tissues. Furthermore, hormone levels regulate components of innate and adaptive immunity (Klein and Flanagan, 2016) and thus are critically involved in some immune-mediated diseases, including Sjögren syndrome, systemic lupus erythematosus, thyroid diseases (such as Hashimoto thyroiditis and Graves disease), scleroderma, and myasthenia gravis, with significantly more women afflicted than men. Cardiovascular diseases, such as stroke, have been found to less likely occur in women before menopause. However, after menopause, the incidence of stroke in women surpasses that of men (Haast et al., 2012).

Based on what has been discussed, we can make some important conclusions about the concept of sexual differentiation of the brain. First, the sex chromosomes X and Y carry multiple genes that initiate sexual differentiation. Second, the signals that act directly on brain cells to cause sexual differentiation are not just triggered by gonadal hormones, but also by other factors, such as gene products encoded by genes and areas downstream the genes of the sex chromosomes. Third, different brain regions have different programs of response to the sex-specific signals, involving regional cell type–specific responses, cell-to-cell communication, effects mediated by membrane and nuclear hormone receptors, local steroid synthesis, and compensatory sex-specific effects that antagonize each other and reduce sex differences in phenotype. Changes in gonadal hormone levels over the lifespan and other dynamic changes are likely to enhance or suppress sex differences over time.

### Genetics and hormones in non-binary gender identities

Recent advances in the field of gender identity development have shed light on the intricate interplay between genetics and hormones in non-binary groups. These insights may help elucidate some of the intriguing distinctions between cisgender individuals and those with non-binary identities concerning brain structure and cognitive abilities (Dotto, 2019). Total brain volume differences exist between cisgender men and women, and similar volumes have been observed in non-binary individuals aligned with their assigned gender at birth (Savic and Arver, 2011; Rametti et al., 2011a; Hahn et al., 2015; Hoekzema et al., 2015). Cortical thickness, another aspect of brain anatomy, displays variations between cisgender men and women, with non-binary individuals showing patterns reflective of their gender identity rather than biological sex (Luders et al., 2006a; Sowell et al., 2007). Furthermore, studies on white matter microstructure and brain activation patterns during cognitive tasks have hinted at the influence of gender identity on brain function. While some investigations have demonstrated differences in brain activation between non-binary individuals and control groups during certain tasks, results have been mixed across studies (Rametti et al., 2011a,b; Rametti et al., 2012; Kranz et al., 2014).

Genetic factors have been explored in relation to gender identity development, with some evidence pointing to specific gene polymorphisms associated with non-binary identities. However, the interaction between genetic factors and environmental influences remains complex. In the literature, there have been reports of multiple non-binary individuals within a single family (Green, 2000), as well as a few cases involving twins (Hyde and Kenna, 1977; Garden and Rothery, 1992; Sadeghi and Fakhrai, 2000; Segal, 2006). To provide additional support for the hypothesis of genetic involvement in the development of gender incongruence, a review of twin case studies has unveiled a higher concordance for gender incongruence in monozygotic twins when compared to their dizygotic counterparts (Heylens et al., 2012). More recently, a study was conducted on a large sample of transwomen and control males to evaluate several candidate genes (Foreman et al., 2019). The authors found a significant association between gender incongruence and estrogen receptor alpha (ERα), SRD5A2, and STS alleles, as well as ERα and SULT2A1 genotypes. These genetic variants could be functional and influence estrogen signaling. Furthermore, inactivating mutations of the androgen receptor gene, or of the gene encoding for the enzyme responsible for testosterone modification (5α-reductase) have been identified as key factors influencing individuals’ gender phenotype. These mutations can lead to non-binary or female phenotypes even in individuals with an XY genotype and testosterone-producing testicles.

On the hormone side, prenatal hormone exposure has emerged as a potential contributor to gender identity development. Studies have examined the impact of prenatal androgen exposure on the general population using indirect measures like otoacoustic emissions (OAEs), which is the weak sound produced by the auditory transduction apparatus of the inner ear. OAEs differ between males and females, with males exhibiting weaker OAEs than females, a distinction that persists throughout life (McFadden and Pasanen, 1998). Transwomen show more female-typical OAE patterns, supporting the theory that they experienced lower androgen exposure during early development compared to control boys (Burke et al., 2014). Additionally, prenatal exposure to anticonvulsants, which may interfere with sex hormone metabolism, was associated with the development of gender incongruence (Dessens et al., 1999). Furthermore, in non-binary individuals, the administration of gender-affirming hormonal treatments has been associated with changes in brain volume and ventricle dimensions (Pol et al., 2006), as well as cortical thickness and subcortical volumes (Zubiaurre-Elorza et al., 2014), revealing the plasticity of the brain in response to hormonal fluctuations.

In conclusion, recent advances reveal a complex interplay between genetics and hormones in non-binary groups, impacting brain structure and cognitive abilities. Genetic factors, such as specific gene polymorphisms and inactivation mutations, suggest a role in gender identity development. On the other hand, hormone exposure also seems to influence this process. A better understanding of these connections will deepen our comprehension of gender identity development’s multifaceted nature.

## Brain macroanatomy and global differences

Sex differences in the brain are found both at the macro and micro level in the brain (Table 1). The most consistent macroscopic observation is that men have an on average larger total brain volume than women. The male brain is about 11% larger than the female brain, which is explained in part by larger body dimensions in men (Paus, 2010). In another study, the brain was approximated to be 8–15% larger in males compared to females (Ruigrok et al., 2014).

**Table 1:** A summary table of brain structures where sex differences have been reported.

Brain structure	Sex difference	Study
Macroanatomy		
Overall brain volume	The male brain is about 8–15% larger than the female brain.	Green (2000) and Kranz et al. (2014)
Brain raw volumes and raw surface areas	Females have thicker cortices.	Garden and Rothery (1992)
Corpus callosum	Males have larger average size.	Pol et al. (2006)
Connectivity	Females show stronger connectivity default mode network (DMN). Males show stronger connectivity in unimodal sensorimotor cortices as well as high-level cortical areas in the rostral lateral prefrontal cortex.	Garden and Rothery (1992)
Gray and white matter	Regional cortical gray matter volumes have a peak size occurring one to three years earlier in females. There are sex differences in white matter microstructure, with females showing lower directionality and higher tract complexity of white matter.	Bishop and Wahlsten (1997), Giedd et al. (1997), and Sullivan et al. (2021)
Microanatomy		
Limbic system	Males show higher densities in the left side of the limbic system, while females show higher densities in the right side of the limbic system.	Green (2000)
Cingulate gyrus	Males show higher densities in left posterior cingulate gyrus, while females show higher densities in anterior cingulate gyrus.	Green (2000)
Insula	Males have larger insula until about 12 years of age, after which the effect reverses.	Ingalhalikar et al. (2014)
Planum temporale and Sylvian fissure	Larger and longer in males compared to females.	Ficek et al. (2021)
Superior temporal cortex and Broca’s area	Larger in females.	Ficek et al. (2021)
Amygdala and hypothalamus	Larger in males.	Allen et al. (1991)
Caudate and hippocampus	Larger in females.	Amft et al. (2015)

All studies were conducted on human subjects.

According to a recent study in over 5,000 participants from United Kingdom Biobank (UKB), males have higher brain raw volumes and raw surface areas, whereas females have thicker cortices (Ritchie et al., 2018). A meta-analysis of sex differences in human brain structure found sustained differences in overall brain volumes between males and females (Ruigrok et al., 2014). The results showed that on average males had larger intracranial volume, total brain volume, cerebrum, gray matter, white matter, cerebrospinal fluid (CSF), and cerebellum absolute volumes than females. It has also been reported by earlier studies that several major brain subdivisions, including the hemispheres, frontal and temporal lobes, left parietal lobe, insula and cerebellum show differences between sexes and are significantly larger in men compared with women. However, the proportional sizes of individual regions in relation to total hemisphere volume seem to be similar (Allen et al., 2002). Other studies have also indicated that while men have greater brain volume (Gur et al., 1999), greater CSF volume or lateral ventricles (Giedd et al., 1997; Gur et al., 1999), and greater sulcal volume (Gur et al., 1999) compared with women, ventricular volumes (Erdogan et al., 2004) and intracranial areas corrected for differences in cranial size do not vary between sexes (Agartz et al., 1992).

Given that men usually have larger brains than women, some possible compensatory mechanisms in smaller female brains have been suggested that might have occurred during human evolution (Luders et al., 2004). Those compensatory mechanisms can be related to three main features: cortical thickness (Clayton, 2018), cortical convolution (Heidari et al., 2017) and cortical surface area (Bielsky et al., 2005). Studies have revealed significantly thicker cortices in women than men (Im et al., 2006) with temporal regions being least different in thickness. This was even confirmed by a third recently published study (Ritchie et al., 2018). According to this later study, females have a thicker cortex across almost the entire brain. The only area where males showed a statistically significantly thicker cortex was the right (but not left) insula. Studies of cortical convolution have observed a greater cortical complexity in female brains in the frontal and parietal lobes (Luders et al., 2004). The observations of previous outcomes of greater cortical complexity in frontal and parietal regions in female brains have been confirmed by another study that in addition has detected more pronounced female convolutions in temporal and occipital cortices as well. Finally, sex differences with respect to the cortical surface area have also been reported in the same study, but the results are inconsistent. The surface areas of the cortices were larger in females compared to males (Luders et al., 2006b). However, a recent study produced results contradicting these previous findings, reporting sex differences in surface area favoring males (Ritchie et al., 2018).

Since adult males have a larger average brain size, they also have larger average size of the entire corpus callosum (CC) (Bishop and Wahlsten, 1997). The CC is a brain structure, which has frequently been studied in the context of questions related to aging, addiction, intelligence, hemispheric asymmetry, and sex differences (Sullivan et al., 2021). The size of the CC correlates with the brain size in both sexes (Jäncke et al., 2015). Although sex differences in multitasking do not seem to be clear (Lui et al., 2021), it is often claimed that women have an advantage in multitasking compared to men which was recently supported by an article using diffusion tensor imaging (DTI) and graph theoretical analyses (Ingalhalikar et al., 2014). The authors identified better and more efficient interhemispheric wiring via the CC in females, while males had greater within-hemispheric connectivity. Earlier studies have claimed that women have larger CCs than expected for their brain size (Allen et al., 1991; Davatzikos and Resnick, 1998), which could indicate that women may have an overproportionally strong interhemispheric connectivity, which would explain the female superiority in multitasking. Studies have further shown sex differences in connectivity in several regions in male brains, including the CC, the anterior cingulate cortex, the insula, the orbitofrontal cortex and the periaqueductal gray (Sacher et al., 2013).

Resting-state functional magnetic resonance imaging (fMRI) analyses have revealed increased connectivity in females in posterior regions but decreased connectivity in anterior regions of the default mode network (DMN), which is a set of widely distributed brain regions in the parietal, temporal, and frontal cortex. The DMN is often active when an individual is at rest and shows reductions in activity during attention-demanding tasks (Ficek et al., 2021). On the other hand, males show stronger connectivity in unimodal sensorimotor cortices as well as high-level cortical areas in the rostral lateral prefrontal cortex (Ritchie et al., 2018). The higher female connectivity within circuits like the DMN may be particularly important, given that DMN regions are often considered as an important part of the “social brain” (Kennedy and Adolphs, 2012; Amft et al., 2015), which may explain higher average female ability in domains like social cognition (Gur et al., 2012). It was further reported that men exhibit stronger connectivity in the right hemisphere, while women show enhanced connectivity in the left hemisphere (Sacher et al., 2013). However, analyses have failed to detect any significant sex effects with respect to hemispheric differences (Watkins et al., 2001).

Gray and white matter vary by sex in both volume and developmental trajectories. It has been reported that the frontal and parietal lobes attain peak gray matter volume at around age 11 in girls and at the age of 12 in boys (Blakemore, 2012). This was confirmed by longitudinal studies indicating that regional cortical gray matter volumes have a peak size occurring one to three years earlier in females (Giedd et al., 2012). Earlier studies indicated that women have a higher percentage of gray matter, whereas men have a higher percentage of white matter and CSF (Gur et al., 1999). However, compared to women, men have consistently higher gray/white matter ratio in frontal, temporal, parietal, and occipital lobes, and even in cingulate gyrus and insula (Allen et al., 2003; Haier et al., 2005). It was also shown for the first time that while gray matter volume and cortical thickness generally decrease with age, gray matter density increases (Gennatas et al., 2017). Regarding white matter microstructure, a recently published study based on data from UKB participants focused on two white matter microstructural properties that had previously been shown to be different between males and females (Ritchie et al., 2018). The first is fractional anisotropy, an index of the directionality of water diffusion through the white matter, while the second is orientation dispersion, a neurite orientation dispersion and density imaging measure of white matter tract complexity. According to the study, females showed lower directionality and higher tract complexity compared to men. Sex differences in white matter microstructure were also revealed in the CC and the cingulate cortex, suggesting differences in myelination between male and female (Sacher et al., 2013).

## Brain microanatomy and regional differences

Although, regional differences in brain volume and structure are less consistent, they do exist and have been reported in many areas (Table 1). Sex dependent differences are seen both on the cortical and subcortical level of the brain. Size differences in smaller brain structures (Goldstein et al., 2001) are important for normal behaviors and diseases and may reflect the disease pattern in men and women.

### Regional cortex

Regional volume and density analyses have been mainly performed for areas that are part of the limbic and language systems and indicate a potential lateral asymmetry in sex differences (Ruigrok et al., 2014). Volume increases in males are mostly in bilateral limbic areas and left posterior cingulate gyrus, whereas higher densities are mostly limited to the left side of the limbic system. On the other hand, larger volumes in females are strongly pronounced in areas of the right hemisphere related to language and in several limbic structures such as the right insular cortex and anterior cingulate gyrus (Ruigrok et al., 2014). Subregional analyses were conducted in a recent meta-analysis (Ruigrok et al., 2014), which found greater volume for females in areas such as the thalamus, the anterior cingulate gyrus, and the lateral occipital cortex. In contrast, a recent study characterized by a more detailed analysis found no sex-specific differences in brain subregions (Ritchie et al., 2018). The same study showed that sex specific differences with higher volume in males appeared to be largest in brain regions linked to emotion and decision-making, such as the bilateral orbitofrontal cortex, the bilateral insula, and the left isthmus of the cingulate gyrus (Craig, 2009; Tang et al., 2010). Sex specific differences were highest in the insula which is consistent with findings in a recent large-scale study performed in children and adolescents (Gennatas et al., 2017). The insula and the right fusiform gyrus retain their substantial sex difference into later life. Furthermore, the planum temporale and Sylvian fissure have been found to be larger and longer in males compared to females. In contrast, the volumes of the superior temporal cortex and Broca’s area were significantly larger in females (Harasty et al., 1997).

### Subcortical regions

Some of the structures commonly reported to differ between sexes include the caudate nucleus, hippocampus, amygdala, and hypothalamus. Men tend to have a larger amygdala and hypothalamus (Goldstein et al., 2001), while women have a larger caudate and hippocampus (Filipek et al., 1994). Sex differences emerge mainly across puberty leading to an increase of the hippocampal volume in females only, and to increases in amygdala volume during puberty in males only (Neufang et al., 2009). This suggests that sex hormones play a prominent role for the structural differentiation in the brain. The hippocampus, which is strongly associated with learning and memory, is evidently sexually diffentiated in its structure and function. Differences exist in a variety of ways, such as size, anatomical structure, neurochemical make-up and reactivity to stressful situations (Goldstein et al., 2001). The amygdala is another brain region that differs between sexes. Previous studies described a sex-related functional lateralization in the amygdala (Domes et al., 2010). The medial nucleus of the amygdala is since a long time known to be sexually differentiated (Cooke and Woolley, 2005). Other studies have provided evidence for a sex differences in almost all of the amygdaloid nuclei (Ziabreva et al., 2003). While a meta-analysis (Marwha et al., 2017) did not detect strong sex dependent differences in the amygdala after correction for total brain volume, another more recent study showed that the amygdala was significantly, but modestly, larger in males even after adjusting for total brain volume (Ritchie et al., 2018). Overall, results regarding the sexually differentiated nature of the human amygdala and hippocampus are still conflicting (Tan et al., 2016; Marwha et al., 2017).

### Sex differences on the cellular level

A rapidly growing body of evidence has revealed sex differences in many fundamental cellular processes, and demonstrated that these differences play a role in shaping the ultimate size and structure of tissues, as well as in determining the dimensions, anatomical characteristics, and cellular composition of organs. Within vertebrates, including humans, sex differences have been observed in various organs such as the brain, heart, fat, pancreas, kidney, and lungs (Mank and Rideout, 2021). While the mechanisms behind these differences remain incompletely understood, distinct phenotypic variances between males and females have been noted. For instance, the higher number of pancreatic β-cells in women is associated with greater glucose-stimulated insulin secretion compared to men (Basu et al., 2006; Horie et al., 2018). Exploration of the cellular level also unveils sex differences in extracellular matrix (ECM) production and remodeling, particularly involving matrix metalloproteinases (MMPs) and their tissue inhibitors (TIMPs). In valvular interstitial cells (VICs), females generally exhibit higher MMP expression and production compared to males, while male VICs demonstrate elevated collagenase and gelatinase activity (Simon et al., 2023). In neonatal rats, there is a higher proliferation in the CA1 and CA3 regions of the hippocampus in males, potentially contributing to a male-biased increase in neuron number. A sex-specific proliferation occurs in the CNS across many species, from rom flies and worms to fish (Taylor and Truman, 1992; DeWulf and Bottjer, 2002; Ampatzis and Dermon, 2007). In mouse brains, sex differences are found in number, distribution, or projections of progesterone receptor (PR)-expressing neurons. Ablation of PR-expressing neurons in the ventromedial hypothalamus diminishes sexual receptivity in female mice and reduces mating and aggression in male mice (Yang et al., 2013). Finally, sex-biased gene expression has been found across 45 common tissues. Male-biased genes were more frequent in skin, skeletal muscle, and cingulate cortex tissues, while female-biased genes dominated the liver, heart, skin, and skeletal muscle. While sex chromosomes held a higher proportion of such genes, 90 percent were mapped to autosomes globally. Male-biased genes exceeded female-biased genes in number and distribution across tissues. The female-biased genes were associated with obesity, muscular diseases, and cardiomyopathy (Guo et al., 2016; Kassam et al., 2019). In this complex landscape of cellular-scale sex differences, extensive investigation is needed to fully comprehend their implications across development, physiology, and behavior.

### Brain metabolism

Several studies suggest significant differences between males and females in functional aspects of brain metabolism and cerebral blood flow. Some early studies reported that women consistently have higher global cerebral blood flow compared with men during rest (Devous et al., 1986; Erdogan et al., 2004) and cognitive activity (Jones et al., 1998; Slosman et al., 2001). The results were confirmed in later studies which showed that females have increased resting state global cerebral blood flow (Gur and Gur, 1990; Ragland et al., 2000). Regionally, it has been noted that males have a more strongly lateralized cerebral blood flow in frontal regions, in the right hemisphere (Rodriguez et al., 1988), while females have a higher regional cerebral blood flow than males in mid-temporal regions (Ragland et al., 2000). These findings may be important for the distribution of psychotropic drugs in the brain. The differences in blood flow may explain why some drugs used in neuropsychiatry are more effective in women as compared to men (Staley et al., 2006). The cerebral metabolic rate of glucose utilization (CMRglu) tends to be higher in women than in men (Baxter et al., 1987), particularly in the orbital frontal area (Andreason et al., 1994). Interestingly, regional CMRglu varies significantly during the menstrual cycle, suggesting hormonal influences on brain glucose metabolism (Reiman et al., 1996). However, males demonstrate a higher cerebral glucose metabolism in other regions, e.g., in the cerebellum, basal ganglia, and brainstem. Sex differences in metabolism may be confuted by the inverse correlation between global CMRglu and brain size leading to higher CMRglu in individuals with smaller brains (Hatazawa et al., 1987; Yoshii et al., 1988).

## Neurochemistry

Differences between men and women exist even on a neurochemical level and may contribute to sex differences in a wide variety of behavioral traits and in the risk to develop neuropsychiatric diseases (Table 2), which is further discussed later in this review. Sex differences occur in a wide array of neurotransmitter systems, mainly in the opioid, dopaminergic (DA), serotonergic (5-HT), cholinergic (ACh) and gamma-aminobutyric acid (GABA)-ergic systems, and affect neurotransmitter levels, receptor and transporter activity and expression. It has been shown that women have significantly higher levels of monoamine oxidase in several brain regions compared to men (Robinson et al., 1977). In a more recent study on rats, the response to stress of the monoamine-rich locus coeruleus in rats was sexually differentiated (Curtis et al., 2006). The study compared locus coeruleus responses to stress in female and male rats under different hormonal conditions and found that the stress-related hormone CRF was 10–30 fold more potent in activating locus coeruleus neurons in female than in male rats. This may explain the increased vulnerability and higher prevalence of stress-related psychiatric disorders in females than in males, particularly depression (Carter-Snell and Hegadoren, 2003; Kuehner, 2003).

**Table 2:** A summary table of neurotransmitters where sex differences have been reported.

Neurotransmitter	Sex difference	Putative disease association
Serotonergic (5-HT) system	Females have higher whole blood 5-HT levels (Gur and Gur, 1990), higher 5-HT transporter availability in the diencephalon and brainstem (Rodriguez et al., 1988), and higher 5-HT1A receptor numbers than males (Baxter et al., 1987; Staley et al., 2006). Males synthesize serotonin significantly faster than females (Andreason et al., 1994).	A wide range of psychiatric disorders, including, depression, anxiety and antisocial personality disorder (Hatazawa et al., 1987; Reiman et al., 1996).
Dopaminergic (DA) system	Females show a higher presynaptic dopaminergic synthesis in the striatum than age-matched males (Robinson et al., 1977). DA transporter, which regulates synaptic DA availability, is higher expressed in the caudate nucleus in females (Curtis et al., 2006).	Anxiety, depression, schizophrenia, addiction, and attention deficit hyperactivity disorder (Kuehner, 2003). DA transporter expression has been positively correlated with learning performance (Carter-Snell and Hegadoren, 2003).
Cholinergic (ACh) system	Females show higher frontal cortex cholinergic activity, while males have higher activity in the hippocampus (Proebstl et al., 2019).	Nicotine addiction (Weiss et al., 2005).
Gamma-aminobutyric acid (GABA)-ergic	Females have higher cortical GABA levels than males (Parsey et al., 2002).	Seizures and movement disorders (Löscher et al., 1987, in rats).
Opoid system	Sex differences exist in opioid affinity, receptor density, signal transduction, and tolerance development (Moen and Lee, 2021; Seeman and Lang, 1990).	Analgesia (Seeman and Lang, 1990). Blood pressure (Cruz and Rodríguez-Manzo, 2000, in rats).

Serotonin is one of the most abundant monoamine neurotransmitters in the brain. Women have higher whole blood 5-HT levels than men (Ortiz et al., 1988), which appears to be genetically determined (Weiss et al., 2005). Healthy women also have higher 5-HT transporter availability in the diencephalon and brainstem compared with men (Proebstl et al., 2019) and higher 5-HT1A receptor numbers than men in certain brain regions (Arango et al., 1995; Parsey et al., 2002). On the other hand, men synthesize serotonin significantly faster than women and reach 52% higher synthesis mean rates (Nishizawa et al., 1997). A rich body of literature demonstrates the involvement of 5-HT in a wide range of disorders. In fact, it appears that all psychiatric disorders are related to 5-HT dysfunctions, and many, if not all, psychotropic drugs used to treat psychopathological conditions interfere, to varying degrees, with the 5-HT system (Deakin, 2003; Davies et al., 2010). However, concerning depression, which is often widely associated with the 5-HT system, recent research has not provided convincing evidence linking depression to lower serotonin concentrations or activity (Moncrieff et al., 2022).

Dopaminergic function is also enhanced in women compared to men. Studies suggest that healthy premenopausal women show a higher presynaptic dopaminergic synthesis in the striatum than age-matched men (Laakso et al., 2002). The DA transporter, which regulates synaptic DA availability, is higher expressed in the caudate nucleus in women compared with men. However, sex differences in D2 receptors are inconsistent (Kaasinen et al., 2001) and D2 receptor availability may vary with fluctuations in sex hormones across the menstrual cycle. DA transporter expression has been positively correlated with learning performance (Mozley et al., 2001). Sex differences in the dopaminergic system have been linked to sex differences in the incidence and prevalence of neuropsychiatric disorders such as anxiety, depression, schizophrenia, addiction, and attention deficit hyperactivity disorder (Williams et al., 2021). Higher dopaminergic tone may also be the reason for a stronger effect of antidopaminergic drugs in women than men (Seeman and Lang, 1990). Furthermore, there is evidence for sex differences in the nicotinic acetylcholine receptor system and in the dopaminergic system in response to nicotine administration and tobacco smoking. Men show a rapid increase in dopamine levels in the ventral striatum and the upregulation of the β2-subunit containing nicotinic acetylcholine receptor (β2-nAChRs) in the cortex and striatum in response to tobacco smoking compared to women (Verplaetse et al., 2018). In a recent review from 2018, it was reported that sex hormones exert trophic effects on the cholinergic system, and that females show higher frontal cortex cholinergic activity whereas males have higher activity in the hippocampus (Giacobini and Pepeu, 2018). Sex differences in the nicotinic acetylcholine system may even underlie sex-related differences in nicotine addiction (Verplaetse et al., 2018; Moen and Lee, 2021).

Differences between men and women have been also reported for GABA. GABA neurotransmission seems to be tightly regulated by the menstrual cycle (Gingnell et al., 2013). MRI studies have shown that women have higher cortical GABA levels than men (Sanacora et al., 1999). Sex differences in GABA neurotransmission may contribute to differences in the incidence of seizures and movement disorders in sexes. In certain experimental models of epilepsy, sex differences in the susceptibility to certain types of seizures, seizure-induced damage, or response to antiepileptics have been reported (Tan and Tan, 2001; Galanopoulou et al., 2003; Kalkbrenner and Standley, 2003). Epilepsy is more common in men than in women (Kotsopoulos et al., 2002). One of the brain areas that has been implicated in the higher susceptibility of males to seizures is the substantia nigra reticulata, which is involved in the control of movement (Garant and Gale, 1983; Löscher et al., 1987). A higher male incidence has also been reported for other diseases, including Tourette syndrome (Freeman et al., 2000) and Parkinson’s disease (Bower et al., 2000), which have been linked to an underlying dysfunction of the substantia nigra reticulata.

Sex differences in opioid peptides and in their analgesic effectiveness have been documented (Craft, 2003). Multiple mechanisms underlying sex differences in opioid analgesia are suggested, including both pharmacokinetics and pharmacodynamics. Sex differences in opioid analgesia can be explained by, for example, a different affinity of opioids for opioid receptors, differences in opioid receptor density, or differences in the signal transduction by opioid receptors (Craft, 2003). There is evidence for sex differences in the density and distribution of opioid receptors and endogenous opioid peptides in the rodent hypothalamus. Moreover, there are indications that opioid receptors and opioid peptide levels can be modulated by gonadal steroid hormones, particularly estrogen, at least in some areas of the brain (Maggi et al., 1991). In addition, a different capacity of opioid receptor binding in several brain regions in men compared to women, including the amygdala and thalamus, has been detected (Zubieta et al., 1999). Several studies in rodents have reported sex differences in the development of opioid tolerance, such that males and females may develop morphine analgesic tolerance at different rates (Kasson and George, 1984; Craft et al., 1999). Besides analgesia, other examples of sex differences in the effect of opioids have been observed, including blood pressure (Cruz and Rodríguez-Manzo, 2000), opioid-induced changes in respiration (Dahan et al., 1998), body temperature (Kest et al., 2000), locomotor activity (Stewart and Rodaros, 1999) and food intake (Marrazzi et al., 1996). Understanding how biologic factors such as how sex influence the opioid effects will enable us to use existing opioid pharmacotherapies more appropriately and efficiently, and perhaps to develop better opioid-based medicines in the future.

There are interactions between sex hormones and important neurotransmitters, such as serotonin, dopamine, GABA and glutamate, which partly explain sex dependent differences, e.g., in neuropsychiatric diseases. Depending on the neurotransmitter system, sex hormones such as estrogen and progesterone can exhibit facilitative, excitatory, and suppressive effects on neurotransmission. For example, progesterone suppresses the excitatory glutamate response (Hausmann and Güntürkün, 2000) and facilitates GABAergic neurotransmission through its action at GABAA receptors (Van Wingen et al., 2008). In contrast, estrogen has been shown to facilitate glutamate transmission (Smith and Woolley, 2004) and to suppress GABA inhibitory inputs. Furthermore, it is well established that estrogens modulate dopamine signaling in the dorsal striatum (Bazzett and Becker, 1994). Estrogen also promotes dopamine release in the striatum, which is thought to be mediated by the inhibitory effect of estrogen on GABA release, as dopamine terminals are influenced by GABAergic inputs. Thus, a decrease in inhibitory tone might facilitate DA release. Estrogen seems also to have an impact on the serotonergic system, thereby exerting effects on cognition and mood (Epperson et al., 2012). Estrogen can even increase serotonin levels and decrease 5-HT reuptake (Koldzic-Zivanovic et al., 2004), which allows 5-HT to stay longer in the synaptic cleft and to exhibit prolonged effects on postsynaptic receptors. Variations in hormone levels across the human lifespan may be particularly significant for the probability of developing certain mood disorder and neurodegenerative pathologies. Increased knowledge about the interplay between gonadal steroid hormones and different neurotransmitter systems may contribute to a better understanding of the etiology of neuropsychiatric diseases that display a relevant sex difference, such as Alzheimer’s disease and depression and will help to develop better preventive and more effective treatment strategies.

## Cognition

The topic of sex differences in cognitive abilities continues to generate interest not only for social scientists but also for the general public and media (Halpern and LaMay, 2000; Reilly, 2012). A better understanding of morphological and functional differences may help explaining several behavioral sex differences (Zell et al., 2015; Gur and Gur, 2017). However, directly linking cognitive performance to biological factors is overly simplistic due to its intricate susceptibility to various variables, including societal influences, culture, gender, self-perception, and hormones, all of which can significantly influence the outcomes. Evidence demonstrates that the extent of sex differences in cognitive performance among middle-aged and older populations across Europe systematically varies across cognitive tasks, birth cohorts, and regions. Furthermore, it highlights a correlation between the cognitive performance exhibited later in life and the living conditions as well as educational opportunities individuals were exposed to during their formative years (Weber et al., 2014).

Although sex differences are small in many psychological functions, they still exist in several domains. To start with women, they tend to perform better in verbal and memory tasks. More specifically, women appear to be better spellers and to have slightly better verbal ‘fluency’ (Kimura, 1999). Women’s advantage in reading (and writing) may be related to an early advantage in many language-related competencies that facilitate learning to read (Halpern, 2021). Further, reading comprehension might also require more complex underlying social-cognitive processes in which women also have higher capabilities (Geary, 1998), such as perspective taking, “theory of mind,” and social understanding (Hatcher et al., 1990; Bosacki and Astington, 1999). Other behaviors and traits that are more common among women include agreeableness (Costa et al., 2001), neuroticism, which includes personality traits that are characterized by negative emotions such as anxiety, anger, and depression (Schmitt et al., 2008), and self-reported interest in people versus things (Su et al., 2009). On the other hand, males perform on average better in mental rotation tasks (Maeda and Yoon, 2013), and they tend to have better spatial abilities than women (Hampson, 1990). Men also show a higher level of physical aggression (Archer, 2004), and they have traditionally been considered more aggressive than women (Leslie and Wilson, 2020). While women express anxiety and fear in response to provocation (Björkqvist, 2018), men tend to respond with aggressive behavior (Archer, 2004; Björkqvist, 2018). Moreover, men lie behind about 80% of all global homicides (United Nations: Office on Drugs and Crime, 2022).

When it comes to mathematical performance, there are sex differences in mathematics anxiety. Women report greater mathematics anxiety in comparison with men (Goetz et al., 2013; Bieg et al., 2015). In terms of performance, the literature presents conflicting findings. While some studies propose that women tend to score lower than men on mathematics tests in most developed nations (Stoet and Geary, 2013), others emphasize that males and females perform similarly, challenging the stereotype that girls and women lack mathematical ability (Bosacki and Astington, 1999). In addition, the statement that only few women among the top performers in mathematics (Else-Quest et al., 2010) should be interpreted with caution, as multiple factors may account for this gender gap. Today women earn 45% of the undergraduate degrees in mathematics, yet they constitute only 17% of university faculty in mathematics (NSF, 2023). Furthermore, studies suggest a correlation between the level of country specific gender equality and sex differences in mathematics performance (Guiso et al., 2008; Else-Quest et al., 2010). Although this is not yet understood from a neuroscientific view, economically developed and countries with a high level of gender equality show larger sex differences in mathematics anxiety compared to less developed countries (Stoet and Geary, 2013). The greater mathematics anxiety among women may be the reason behind the lower participation of women than men in career paths that involve mathematics (Beilock et al., 2010). There is an underrepresentation of women in Science, Technology, Engineering, and Mathematics (STEM) related fields and as we make progress in gender equality, the sex differences in the pursuit of STEM degrees have paradoxically risen (Stoet and Geary, 2018). Sex differences in patterns of interest may contribute to this phenomenon (Su et al., 2009), although these patterns, too, are shaped by culture. The existence of stereotypes about female inferiority are also of significant concern, as they can discourage women from entering or persisting in STEM careers.

A modest correlation between cerebrum size and intelligence quotient (IQ) within a sex has been reported (Pietschnig et al., 2015). While there is almost unanimous consensus that men and women do not differ in general intelligence (Neisser et al., 1996; Halpern et al., 2007), men and women tend to use different brain areas to achieve a similar IQ. In men, IQ correlates with gray matter volume in the frontal and parietal lobes, whereas in women, IQ correlates with gray matter volume in the frontal lobe and Broca’s area, linked to language use (Haier et al., 2005). Males exhibit greater functional connectivity than females and the differences are mainly present in frontal, parietal, and temporal lobes. Both male and female brains exhibit small-world organization, a concept in neuroscience that describes the brain’s network organization. This concept quantifies how effectively information is exchanged within local neighborhoods, resulting in efficient information segregation and integration at low wiring and energy costs. Male brains are more locally clustered in all lobes of the cerebrum and are more segregated, while female brains have a higher clustering coefficient at the whole brain level and are more integrated (Zhang et al., 2016). Studies have found that males show greater variance in cognitive ability (Lakin, 2013; Iliescu et al., 2016). Greater variance in males has also been documented for psychological characteristics such as personality (Borkenau et al., 2013) and physical traits such as athletic performance (Olds et al., 2006).

All the differences in diverse cognitive abilities need to be scrutinized critically and interpreted with utmost caution. While genetic vulnerability could offer an initial explanation, the sex differences observed across different contexts imply a multifaceted interplay of social, environmental, and genetic elements that shape these disparities over the course of life. Evidence suggests that sex differences in the brain are largely the result of experience because experience changes the brain (Squire, 1992). Both environmental enrichment studies in animals as well as studies in humans indicate that the mammalian brain changes as a consequence of experience (Kempermann et al., 2002; Trachtenberg et al., 2002). Gender-specific environments are being established as early as in infancy, for example through toys, social interactions and behavioral expectations. Discouraging boys from openly expressing emotions, such as crying, and enforcing a “be a man” mentality, along with clothing girls in garments that constrain their movements to conform to gender norms, can significantly influence the maturing brain. The report by Hines and her colleagues (Hines et al., 2016) in this same issue, depicting how girls emulate behaviors based on female role models, serves as evidence that such processes can indeed take place.

While women generally tend to outperform men on most verbal tests and men tend to excel in visual–spatial tasks, it’s important to note that the effect sizes associated with these differences are generally small. This implies that the overlap in the distribution of scores between males and females is more substantial than the disparities between them. Numerous studies have demonstrated that performance in mental rotation is greatly influenced by the practice of spatial tasks, as well as by one’s educational and cultural background (Peters et al., 2006; Hoffman et al., 2011). Furthermore, in terms of self-rating, men consistently rate their spatial abilities significantly higher than those of women, whereas women do not rate their verbal abilities higher to those of men (Weiss et al., 2003). Sex differences have also been reported in the self-estimation of intelligence (Beyer, 1999). A similar picture emerges for mathematical performance, for which a comprehensive meta-analysis encompassing data from 242 studies conducted between 1990 and 2007, involving 1.2 million children and adults, reveals no sex differences in math performance (*d* = 0.05; Lindberg et al., 2010). In addition, large international studies have revealed significant cross-cultural discrepancies in cognitive sex differences, thereby challenging the idea of universal male advantages in mathematics and female advantages in verbal abilities (Else-Quest et al., 2010; Levine et al., 2016).

## Pathology

**Figure 1:**
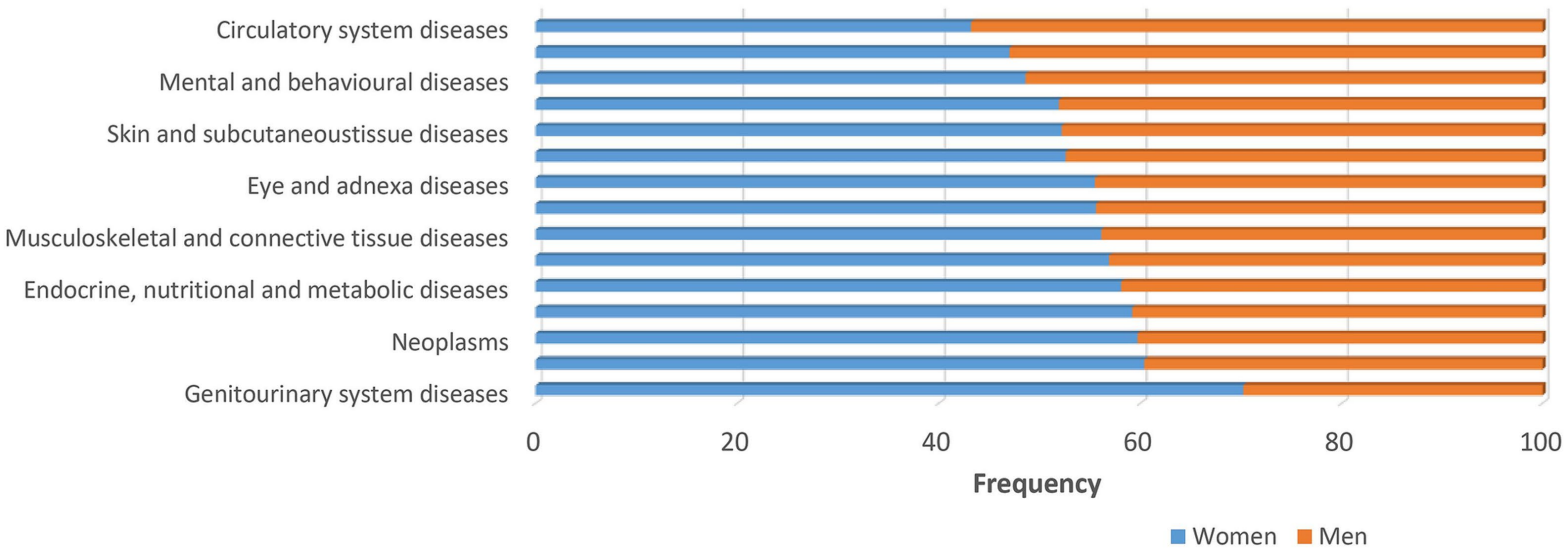
Major disease categories. Major disease categories stratified by sex using data from UK biobank. Sex differences are apparent for many diseases. The frequency for men is generally lower as indicated by the orange bars. The frequency of almost all diseases, except circulatory diseases, respiratory system diseases and mental and behavioral diseases, is higher among women (>50%) as indicated by the blue bars.

**Figure 2:**
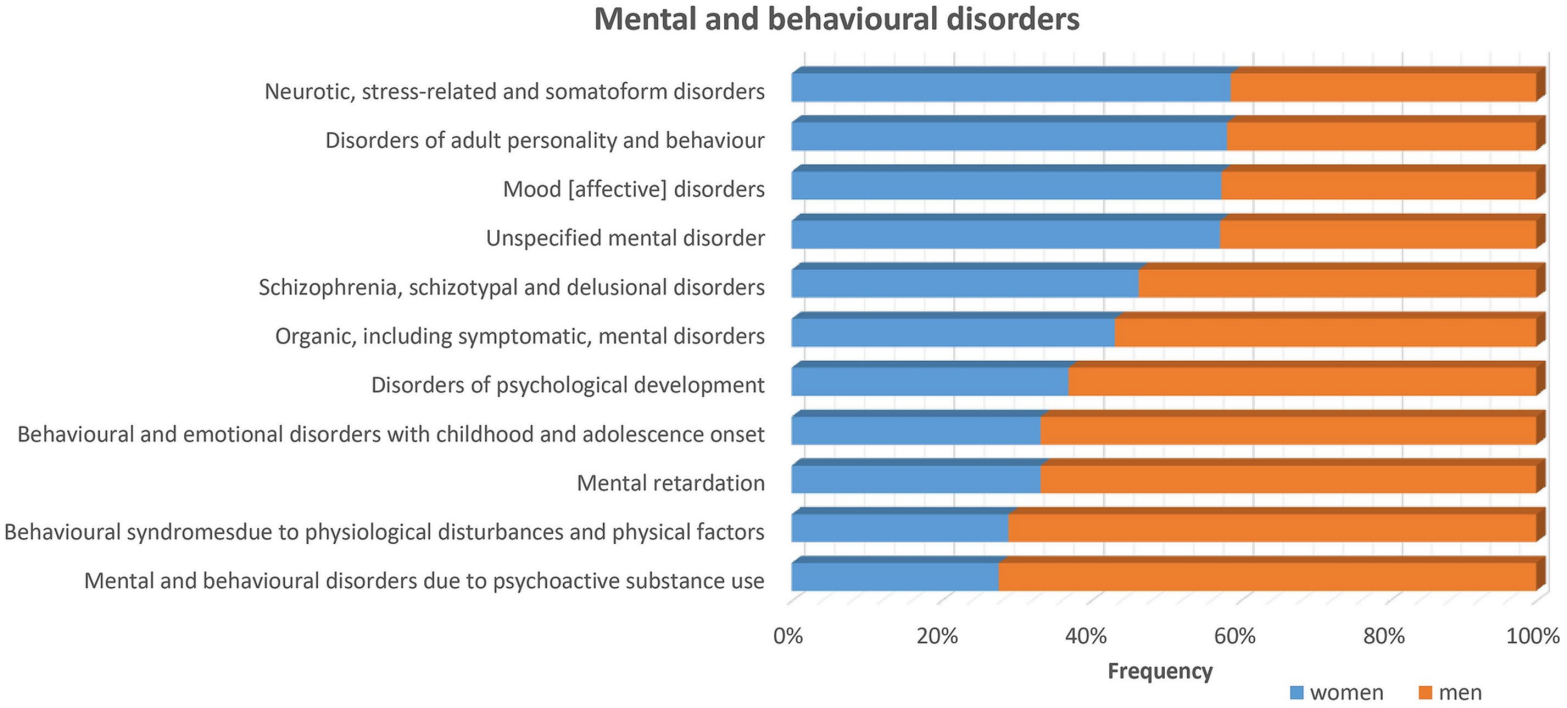
Sex differences in mental and behavioral disorders stratified by sex using data from UK biobank exist for many diseases of the nervous system as listed in the figure. The frequency for most disorders is higher for men (>50%) as indicated by the orange bars. While for some other disorders, i.e., neurotic, stress-related and somatoform disorders, disorders of adult personality and behavior, mood (affective) disorders and unspecified mental disorders, the frequency is higher among women as indicated by the blue bars.

**Figure 3:**
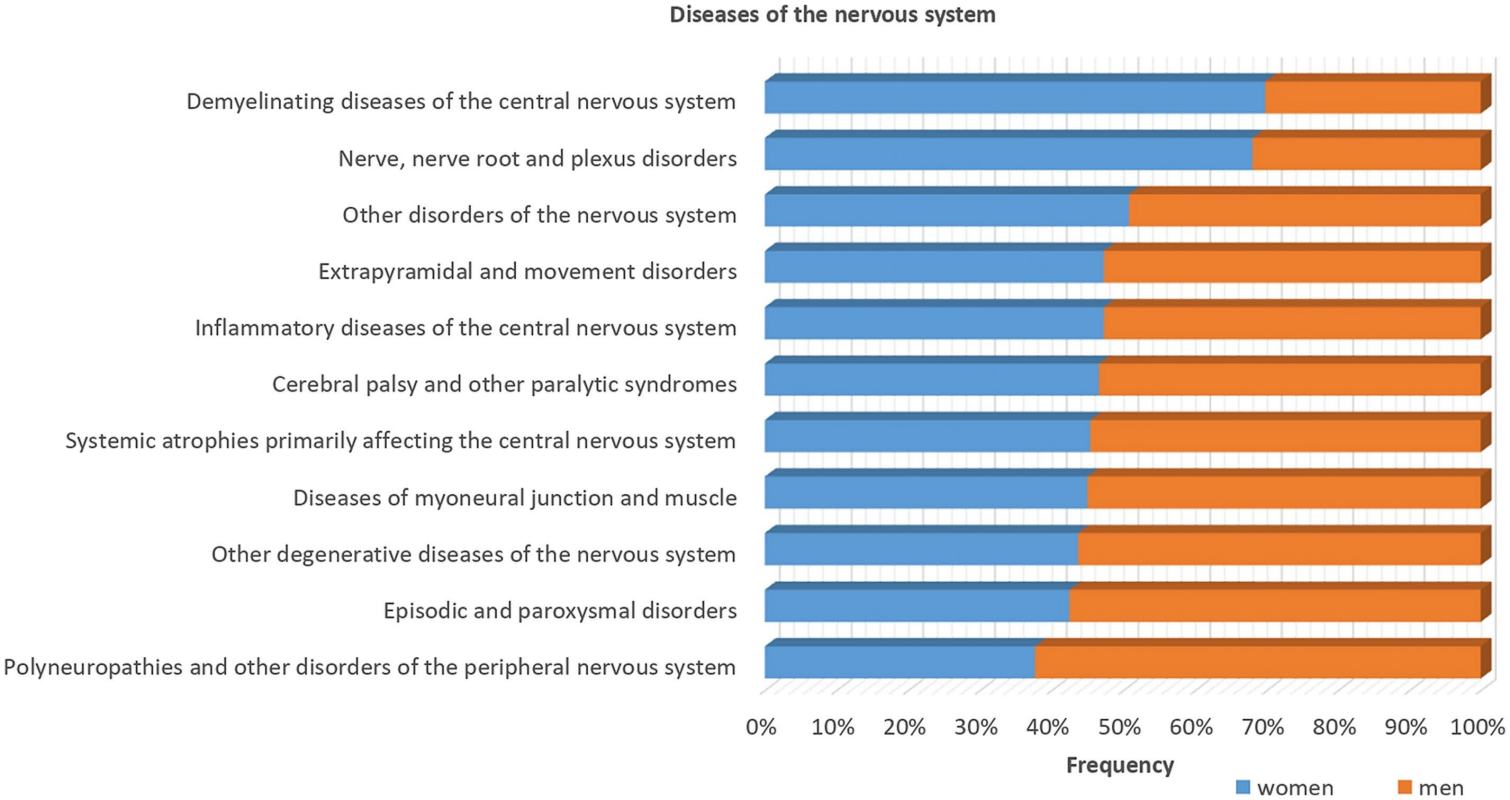
Sex differences in the diseases of the nervous system stratified by sex using data from UK biobank. Most of the diseases show higher frequencies in men (>50%) as indicated by the orange bars, except demyelinating diseases of the central nervous system and nerve, nerve root and plexus disorders, as indicated by the blue bars that are more common among women.

Understanding the biology of sex-dependent brain development and function may help to explain the pathophysiological mechanisms underlying different diseases and may lead to more effective and individually adapted therapeutic interventions (Rutter et al., 2003). In a recent study on sex differences in complex traits in the UKB for a total of 530 traits from around half a million individuals, evidence of sex differences was found for 71 of the traits considered (Bernabeu et al., 2021). There are many examples of neuropsychiatric disorders and other diseases that affect men and women in a sex-specific way with regard to onset, symptomatology, prevalence and severity (Figures 1–3). For instance, males display higher rates of disorders belonging to the autism spectrum. Autism is estimated to be 4 to 7-fold more common in boys than girls (Baron-Cohen et al., 2011). Furthermore, the incidence of developmental language disorders is generally higher in males than females, with dyslexia being three to four times higher in boys (Arnett et al., 2017). ADHD is also significantly more common in males (Nøvik et al., 2006; Munkvold et al., 2011), and boys show more impulsivity, hyperactivity and externalizing relative to girls (Biederman et al., 2002; Gershon, 2002). Men have a higher risk for cardiovascular diseases, such as stroke, than women before menopause. However, after menopause, the incidence of stroke among women surpasses that of men (Haast et al., 2012). A recent study found evidence of a sex-specific genetic burden of risk for hypertension, particularly early-onset hypertension, that is more pronounced in women than men (Kauko et al., 2021). Alcoholism affects men more than women (Ceylan-Isik et al., 2010). Parkinson’s disease is more prevalent in men than women (Georgiev et al., 2017), and sex differences exist also in the prevalence of schizophrenia (Aleman et al., 2003; Bergen et al., 2014).

On the other hand, females show a higher prevalence of major depressive disorder (Gobinath et al., 2017). Anxiety and depression are also more common in females starting from puberty (Hayward and Sanborn, 2002). Anxiety and fear-based disorders affect men and women disproportionately and the mechanisms underlying fear-related disorders are sexually differentiated, according to a recent study that investigated sex differences in fear extinction (Velasco et al., 2019). Moreover, eating disorders are more prevalent in females (Swanson et al., 2011), with differences evolving in adolescence (Giedd et al., 2012). Men and women show differences in eating behavior and there are differential brain responses to food or eating stimuli in various structures. Pathological eating patterns which are more common among females makes women more vulnerable to obesity. It has been suggested that females may be more reactive to visual food stimuli and have greater difficulties deactivating limbic areas of the brain associated with emotional regulation to overcome the motivational drive to eat (Chao et al., 2017; Yeung, 2018). Women are furthermore at higher risk for addiction than men. Women are more sensitive to the reinforcing effects of psychostimulants, such as amphetamine and cocaine, and they show a more rapid progression from initial use to drug dependence (Lynch et al., 2002). There are differences in brain activation in response to drugs between males and females (Kilts et al., 2004), and differences have been discovered even in the responsiveness of dopamine to stimulation by, for example, amphetamine (Becker, 1999). The literature also demonstrates that headache, in particular migraine, is more prevalent in women than men, which is thought to be due the role of sex hormones, especially due to higher estrogen levels in women (Chai et al., 2014). In general, women are strongly overrepresented among patients with chronic pain (Riley et al., 1998). While sex differences in pain sensitivity explain some of the variances (Ruau et al., 2012), some other common pain conditions are tightly connected to female anatomy and physiology, including endometriosis, vulvodynia and menstrual pain.

Providing a clear characterization of neurobiological sex differences is a step toward a better understanding of the differential prevalence in neuropsychiatric disorders. However, a comprehensive perspective requires the consideration of diverse factors, such as age among other variables, to elucidate the underlying sex differences in pathological processes. For instance, a notable illustration lies in the increased susceptibility of females at an advanced age to heightened concentrations of antipsychotics (Castberg et al., 2017). In addition, sociocultural norms and the interpretation of diagnostic criteria are likely underlying factors contributing to some of the evident sex differences in disease prevalence. For instance, reduced help-seeking behavior among men could account for their lower treatment rates for depression (Möller-Leimkühler, 2002), and existing diagnostic guidelines often lead clinicians to under-diagnose ASD in females (Hull et al., 2020). Insights into how and where the brain differs as a function of sex and how sex interacts with other factors, such as age, will enable more targeted examinations into the potential drivers of these disparities. Subsequently, this nuanced understanding will play a pivotal role in refining therapeutic strategies for these conditions, enabling more effective approaches to tackle them.

## Discussion

Our understanding of how the sex chromosomes act as main drivers in the control molecular pathways responsible for the development of sex differences in the brain is rapidly growing. The expression of genes on the X and Y chromosome is crucial for early brain development and mediation of the early phase of sex difference development. The sex chromosome genes control regulatory cascades relating to the expression of other genes encoded in the autosomes. The sex-biased gene expression influences alternative splicing and epigenetic control mechanisms as well as range of hormones and local regulatory factors. It is becoming increasingly evident that the differential splicing and epigenetic control have important contribution to sex-differentiated phenotypes and the establishment and maintenance of sex differences. Different epigenetic mechanisms include DNA methylation, histone modifications, nucleosome repositioning, chromatin accessibility, mechanisms involving non-coding RNA, and RNA and DNA editing. Furthermore, the evidence on how the brain represents an important target for sex hormones and how they act throughout the entire brain of both males and females is also growing. There is a main impact of sex hormones on brain development and plasticity and the hormones exert organizational effects on the brain during neurodevelopment as well as altering intrinsic functions in the brain during lifetime. Future work is warranted to explore in much greater depth the links between sex differences in the brain at multiple points across the lifespan.

Most traits are influenced by one or more genes interacting in complex ways with the environment and while some traits are strongly influenced by genes, other traits are strongly influenced by the environment. The effect of the environment on the biological functions are mediated in many difference ways. For example, molecular inputs to chromatin via cellular metabolism are modifiers of the epigenome (Dai et al., 2020). Different inputs, for example nutrient availability, pharmacological and behavioral treatments are implicated in linking the environment to the maintenance of cellular homeostasis and cell identity depending on the individual genetic composition (Gentner and Leppert, 2019). Many factors modify DNA and histones and exert specific effects on cell biology, systemic physiology and thus basic pathology (Wissink et al., 2019; Dai et al., 2020). Research has explored the epigenetic dynamics within psychiatric disorders and the impact of environmental influences on neurodevelopmental processes (Kofink et al., 2013). However, the origins of these differences, whether they stem from environmental experiences or non-environmental genetic or hormonal factors, remain incompletely understood.

There are some fundamental differences related to the brain anatomy. Males generally show a higher brain weight with higher raw volumes, larger raw surface areas, but thinner cortices than females. While males have a larger average size of the entire CC and greater within-hemispheric connectivity, females have better and more efficient interhemispheric wiring via the CC, which might explain their superiority in multitasking. Other macroscopic differences include larger amounts of gray matter, white matter, CSF, and cerebellum in males than females. Moreover, there is evidence for sex differences in specific brain structures, including the caudate nucleus, hippocampus, amygdala, and hypothalamus, as well as in aspects such as brain metabolism and cerebral blood flow. However, some of these studies show different results for regional volumes dependent on whether a correction for total brain volume has been performed, or not, and further work is needed to promote consensus on the methodological approach. In general, although there are consistent reports and documentation of sex differences, certain studies contend that these differences might not be sufficient to establish a clear sex difference in terms of brain anatomy (Joel et al., 2015). This is primarily due to the extensive overlap in anatomical parameters between men and women. Another crucial aspect is the demonstrated influence of various factors on brain anatomy, such as nutrition, obesity, diet, cultural factors, historical experiences of famine, age, educational background, cardiovascular risk factors, and skill level (Minagar et al., 2000; Münte et al., 2002; Birns et al., 2008; Hedden et al., 2008; Jäncke, 2009; Lupien et al., 2009; Mungas et al., 2009; Weiner et al., 2009; Ho et al., 2010; May, 2011; Braskie et al., 2014; Raji et al., 2014, 2016; De Rooij et al., 2016; Beyer et al., 2017; Kharabian Masouleh et al., 2018).

There is substantial evidence supporting the difference between males and females on a neurochemical level. These differences are of particular interest for explaining the large differences in the prevalence of psychiatric diseases but are also interesting for the differences in basal functions. Many studies have been performed on experimental animal models such as rats and mice and there is substantial evidence that the basic neurochemical networks are highly evolutionary conserved in different mammals (Elphick and Egertová, 2001; Azmitia, 2007; Hoyle, 2011; Gou et al., 2012). Sex differences encompass various neurotransmitter systems including opioid, dopaminergic, serotonergic, cholinergic, and GABAergic systems. Characterization of neurochemical sex differences is very important to understand patterns of differential prevalence and course in neurodevelopmental disorders such as autism spectrum disorder (Baron-Cohen et al., 2011), a variety of psychiatric conditions such as schizophrenia (Aleman et al., 2003), and neurodegenerative disorders such as Alzheimer’s disease (Mazure and Swendsen, 2016). Improving our knowledge about neurochemistry will enhance our ability to explain the pathophysiology of many neuropsychiatric brain disorders that differ between the sexes and ultimately lead to more effective interventions. Also, to realize personalized or precision medicine using pharmacology, it is imperative to understand sex-differentiated disease mechanisms and target drug development with a sex-aware approach. Careful attention to sex differences in preclinical and clinical research before the release of new therapeutics may prevent any growing disparities in health care and optimize therapeutics to perform equally well in males and females.

Males and females show differences in several cognitive and behavioral domains but the correlation with the difference in gene expression, neuroanatomy and neurochemistry is often not well causatively explained although the effect of estrogen and testosterone on many cognitive and behavioral domains are well understood. An important domain to highlight with regard to this aspect is the sex differences in aggression and criminal behavior, as men tend to be more aggressive than women which is reflected in the fact that about 80% of all global homicides are committed by men. Other domains may be less well connected to the neurochemistry or sex hormones. Sex differences in the cognitive domain may have consequences for education and career choices which may have several explanation models. For example, sex differences in mathematics performance and mathematics anxiety may explain the underrepresentation of women in fields such as mathematics and STEM. Interestingly, higher-developed countries with higher living standards show larger sex differences in mathematics. It has been suggested by Halpern (2021) that this finding may be a result of the “Matthew effect” (Ceci and Papierno, 2005), which leads to growing differences when more resources are targeted at improving skills in the areas in which there is a difference. However, it is important to look beyond traditional equality issues and also invest in research in how other factors, such as interest differences, contribute to the sex differences in performance in the different domains.

Finally, the study of sex differences in the brain has gained significant attention and debate within the scientific community. While there are well-established sex differences in certain brain structures and functions, there is also a considerable amount of divergence in the findings, leading to ongoing debates about the nature and significance of these differences. This divergence can be attributed to various factors, including methodological differences, sample sizes, cultural influences, and the complex interplay between biology and environment. One area of divergence pertains to the structural differences between male and female brains. While some studies have reported differences in brain size, the significance of these size differences and their relationship to cognitive abilities remains debated. Sex-associated neural connectivity and wiring differences exist, particularly with regards to front-to-back and interhemispheric connectivity in males and females, respectively. However, the cognitive and behavioral significance of these variations remains unclear and subject to ongoing debate. Research on functional brain activation patterns during tasks also highlights divergences between males and females, wherein specific tasks can trigger differing brain region activation, raising questions about the extent to which these differences reflect inherent cognitive disparities or are influenced by societal factors and individual experiences. Moreover, the role of sex hormones, particularly estrogen and testosterone, in shaping brain development and function adds another layer of complexity to the divergence in findings. Hormonal fluctuations are linked to brain activity and structural changes, but the exact mechanisms and extent of their impact on observed cognitive differences are still under investigation. The brain’s neuroplasticity further complicates the understanding of sex differences as adaptable neural circuits blur the line between inherent differences and learned behaviors. Societal and cultural factors play a significant role in shaping observed sex differences. Traditional gender roles, expectations, and experiences can impact cognitive development and behavior. Teasing apart the effects of biology from those of societal influences is a challenging task and a source of ongoing debate.

In conclusion, while there are undoubtedly well-established sex differences in the brain, the field remains characterized by divergence in findings and ongoing debates. The complex interplay between biology, environment, and culture makes it difficult to untangle the exact causes and implications of these differences. Future research efforts should prioritize rigorous methodologies, large and diverse sample sizes, and interdisciplinary collaboration to advance our understanding of the underlying mechanisms and significance of sex differences in the brain.

## Author contributions

ML: Writing – review & editing, Conceptualization, Data curation, Formal analysis, Methodology, Writing – original draft. JM: Writing – review & editing. OA: Writing – review & editing. GR: Visualization, Writing – review & editing. JD: Writing – review & editing. GA: Writing – review & editing. HS: Conceptualization, Resources, Supervision, Writing – review & editing.

## References

[ref1] Agartz I. Sääf J. Wahlund L. O. Wetterberg L. (1992). Quantitative estimations of cerebrospinal fluid spaces and brain regions in healthy controls using computer-assisted tissue classification of magnetic resonance images: relation to age and sex. Magn. Reson. Imaging 10, 217–226. doi: 10.1016/0730-725X(92)90482-F, PMID: 1564991

[ref2] Aleman A. Kahn R. S. Selten J. P. (2003). Sex differences in the risk of schizophrenia: evidence from meta-analysis. Arch. Gen. Psychiatry 60, 565–571. doi: 10.1001/archpsyc.60.6.565 12796219

[ref3] Allen J. S. Damasio H. Grabowski T. J. (2002). Normal neuroanatomical variation in the human brain: an MRI-volumetric study. Am. J. Phys. Anthropol. 118, 341–358. doi: 10.1002/ajpa.10092, PMID: 12124914

[ref4] Allen J. S. Damasio H. Grabowski T. J. Bruss J. Zhang W. (2003). Sexual dimorphism and asymmetries in the gray-white composition of the human cerebrum. Neuroimage 18, 880–894. doi: 10.1016/S1053-8119(03)00034-X, PMID: 12725764

[ref5] Allen L. S. Richey M. F. Chai Y. M. Gorski R. A. (1991). Sex differences in the corpus callosum of the living human being. J. Neurosci. 11, 933–942. doi: 10.1523/JNEUROSCI.11-04-00933.1991, PMID: 2010816 PMC6575363

[ref6] Amft M. Bzdok D. Laird A. R. Fox P. T. Schilbach L. Eickhoff S. B. (2015). Definition and characterization of an extended social-affective default network. Brain Struct. Funct. 220, 1031–1049. doi: 10.1007/s00429-013-0698-0, PMID: 24399179 PMC4087104

[ref7] Amin Z. Epperson C. N. Constable R. T. Canli T. (2006). Effects of estrogen variation on neural correlates of emotional response inhibition. NeuroImage 32, 457–464. doi: 10.1016/j.neuroimage.2006.03.013, PMID: 16644236

[ref8] Ampatzis K. Dermon C. R. (2007). Sex differences in adult cell proliferation within the zebrafish (*Danio rerio*) cerebellum. Eur. J. Neurosci. 25, 1030–1040. doi: 10.1111/j.1460-9568.2007.05366.x, PMID: 17331199

[ref9] Andreason P. J. Zametkin A. J. Guo A. C. Baldwin P. Cohen R. M. (1994). Gender-related differences in regional cerebral glucose metabolism in normal volunteers. Psychiatry Res. 51, 175–183. doi: 10.1016/0165-1781(94)90037-X, PMID: 8022952

[ref10] Arango V. Underwood M. D. Gubbi A. V. Mann J. J. (1995). Localized alterations in pre- and postsynaptic serotonin binding sites in the ventrolateral prefrontal cortex of suicide victims. Brain Res. 688, 121–133. doi: 10.1016/0006-8993(95)00523-S, PMID: 8542298

[ref11] Archer J. (2004). Sex differences in aggression in Real-world settings: a Meta-analytic review. Rev. Gen. Psychol. 8, 291–322. doi: 10.1037/1089-2680.8.4.291

[ref12] Arnett A. B. Pennington B. F. Peterson R. L. Willcutt E. G. DeFries J. C. Olson R. K. (2017). Explaining the sex difference in dyslexia. J. Child Psychol. Psychiatry 58, 719–727. doi: 10.1111/jcpp.12691, PMID: 28176347 PMC5438271

[ref13] Arnold A. P. Reue K. Eghbali M. Vilain E. Chen X. Ghahramani N.. (2016). The importance of having two X chromosomes. Philos. Trans. R. Soc. Lond. B Biol Sci. 371:20150113. doi: 10.1098/rstb.2015.0113 26833834 PMC4785899

[ref14] Arnold A. P. Rissman E. F. De Vries G. J. (2003). Two perspectives on the origin of sex differences in the brain. Ann. N. Y. Acad. Sci. 1007, 176–188. doi: 10.1196/annals.1286.018 14993052

[ref15] Azmitia E. C. (2007). Serotonin and brain: evolution, neuroplasticity, and homeostasis. Int. Rev. Neurobiol. 77, 31–56. doi: 10.1016/S0074-7742(06)77002-7 17178471

[ref16] Bale T. L. Epperson C. N. (2015). Sex differences and stress across the lifespan. Nat. Neurosci. 18, 1413–1420. doi: 10.1038/nn.4112, PMID: 26404716 PMC4620712

[ref17] Baron-Cohen S. Lombardo M. V. Auyeung B. Ashwin E. Chakrabarti B. Knickmeyer R. (2011). Why are autism spectrum conditions more prevalent in males? PLoS Biol. 9:e1001081. doi: 10.1371/journal.pbio.1001081, PMID: 21695109 PMC3114757

[ref18] Basu R. Dalla Man C. Campioni M. Basu A. Klee G. Toffolo G.. (2006). Effects of age and sex on postprandial glucose metabolism: differences in glucose turnover, insulin secretion, insulin action, and hepatic insulin extraction. Diabetes 55, 2001–2014. doi: 10.2337/db05-1692 16804069

[ref19] Baxter L. R. Mazziotta J. C. Phelps M. E. Selin C. E. Guze B. H. Fairbanks L. (1987). Cerebral glucose metabolic rates in normal human females versus normal males. Psychiatry Res. 21, 237–245. doi: 10.1016/0165-1781(87)90028-x 3498176

[ref20] Bazzett T. J. Becker J. B. (1994). Sex differences in the rapid and acute effects of estrogen on striatal D2 dopamine receptor binding. Brain Res. 637, 163–172. doi: 10.1016/0006-8993(94)91229-7, PMID: 8180794

[ref21] Becker J. B. (1999). Gender differences in dopaminergic function in striatum and nucleus accumbens. Pharmacol. Biochem. Behav. 64, 803–812. doi: 10.1016/S0091-3057(99)00168-9 10593204

[ref22] Beilock S. L. Gunderson E. A. Ramirez G. Levine S. C. (2010). Female teachers’ math anxiety affects girls’ math achievement. Proc. Natl. Acad. Sci. USA 107, 1860–1863. doi: 10.1073/pnas.0910967107, PMID: 20133834 PMC2836676

[ref23] Berchtold N. C. Cribbs D. H. Coleman P. D. Rogers J. Head E. Kim R.. (2008). Gene expression changes in the course of normal brain aging are sexually dimorphic. Proc. Natl. Acad. Sci. USA 105, 15605–15610. doi: 10.1073/pnas.0806883105, PMID: 18832152 PMC2563070

[ref24] Berenbaum S. A. Beltz A. M. (2016). How early hormones shape gender development. Curr. Opin. Behav. Sci. 7, 53–60. doi: 10.1016/j.cobeha.2015.11.011, PMID: 26688827 PMC4681519

[ref25] Bergen S. E. O’Dushlaine C. T. Lee P. H. Fanous A. H. Ruderfer D. M. Ripke S.. (2014). Genetic modifiers and subtypes in schizophrenia: investigations of age at onset, severity, sex and family history. Schizophr. Res. 154, 48–53. doi: 10.1016/j.schres.2014.01.030 24581549 PMC4422643

[ref26] Berman K. F. Schmidt P. J. Rubinow D. R. Danaceau M. A. Van Horn J. D. Esposito G.. (1997). Modulation of cognition-specific cortical activity by gonadal steroids: a positron-emission tomography study in women. Proc. Natl. Acad. Sci. USA 94, 8836–8841. doi: 10.1073/pnas.94.16.8836, PMID: 9238064 PMC23156

[ref27] Bernabeu E. Canela-Xandri O. Rawlik K. Talenti A. Prendergast J. Tenesa A. (2021). Sex differences in genetic architecture in the UK biobank. Nat. Genet. 53, 1283–1289. doi: 10.1038/s41588-021-00912-0, PMID: 34493869

[ref28] Beyer S. (1999). Gender differences in the accuracy of grade expectancies and evaluations. Sex Roles 41, 279–296. doi: 10.1023/A:1018810430105

[ref29] Beyer F. Kharabian Masouleh S. Huntenburg J. M. Lampe L. Luck T. Riedel-Heller S. G.. (2017). Higher body mass index is associated with reduced posterior default mode connectivity in older adults: obesity and the default mode network in aging. Hum. Brain Map. 38, 3502–3515. doi: 10.1002/hbm.23605 PMC6867016 28397392

[ref30] Biederman J. Mick E. Faraone S. V. Braaten E. Doyle A. Spencer T.. (2002). Influence of gender on attention deficit hyperactivity disorder in children referred to a psychiatric clinic. Am. J. Psychiatry 159, 36–42. doi: 10.1176/appi.ajp.159.1.36, PMID: 11772687

[ref31] Bieg M. Goetz T. Wolter I. Hall N. C. (2015). Gender stereotype endorsement differentially predicts girls’ and boys’ trait-state discrepancy in math anxiety. Front. Psychol. 6:1404. doi: 10.3389/fpsyg.2015.01404 26441778 PMC4585180

[ref32] Bielsky I. F. Hu S. B. Young L. J. (2005). Sexual dimorphism in the vasopressin system: lack of an altered behavioral phenotype in female V1a receptor knockout mice. Behav. Brain Res. 164, 132–136. doi: 10.1016/j.bbr.2005.06.005, PMID: 16046007

[ref33] Birns J. Morris R. Jarosz J. Markus H. Kalra L. (2008). Ethnic differences in the cerebrovascular impact of hypertension. Cerebrovasc. Dis. 25, 408–416. doi: 10.1159/000121341, PMID: 18349534

[ref34] Bishop K. M. Wahlsten D. (1997). Sex differences in the human corpus callosum: myth or reality? Neurosci. Biobehav. Rev. 21, 581–601. doi: 10.1016/S0149-7634(96)00049-8 9353793

[ref35] Björkqvist K. (2018). Gender differences in aggression. Curr. Opin. Psychol. 19, 39–42. doi: 10.1016/j.copsyc.2017.03.030 29279220

[ref36] Blakemore S. J. (2012). Imaging brain development: the adolescent brain. NeuroImage 61, 397–406. doi: 10.1016/j.neuroimage.2011.11.080 22178817

[ref37] Blekhman R. Marioni J. C. Zumbo P. Stephens M. Gilad Y. (2010). Sex-specific and lineage-specific alternative splicing in primates. Genome Res. 20, 180–189. doi: 10.1101/gr.099226.109, PMID: 20009012 PMC2813474

[ref38] Borkenau P. McCrae R. R. Terracciano A. (2013). Do men vary more than women in personality? A study in 51 cultures. J. Res. Pers. 47, 135–144. doi: 10.1016/j.jrp.2012.12.001, PMID: 23559686 PMC3612964

[ref39] Bosacki S. Astington J. W. (1999). Theory of mind in preadolescence: relations between social understanding and social competence. Soc. Dev. 8, 237–255. doi: 10.1111/1467-9507.00093

[ref40] Bourdeau V. Deschênes J. Métivier R. Nagai Y. Nguyen D. Bretschneider N.. (2004). Genome-wide identification of high-affinity estrogen response elements in human and mouse. Mol. Endocrinol. 18, 1411–1427. doi: 10.1210/me.2003-0441, PMID: 15001666

[ref41] Bower J. H. Maraganore D. M. McDonnell S. K. Rocca W. A. (2000). Influence of strict, intermediate, and broad diagnostic criteria on the age- and sex-specific incidence of Parkinson’s disease. Mov. Disord. 15, 819–825. doi: 10.1002/1531-8257(200009)15:5<819::AID-MDS1009>3.0.CO;2-P, PMID: 11009185

[ref42] Braskie M. N. Boyle C. P. Rajagopalan P. Gutman B. A. Toga A. W. Raji C. A.. (2014). Physical activity, inflammation, and volume of the aging brain. Neuroscience 273, 199–209. doi: 10.1016/j.neuroscience.2014.05.005, PMID: 24836855 PMC4076831

[ref43] Brennan J. Capel B. (2004). One tissue, two fates: molecular genetic events that underlie testis versus ovary development. Nat. Rev. Genet. 5, 509–521. doi: 10.1038/nrg1381, PMID: 15211353

[ref44] Brinton R. D. Thompson R. F. Foy M. R. Baudry M. Wang J. Finch C. E.. (2008). Progesterone receptors: form and function in brain. Front. Neuroendocrinol. 29, 313–339. doi: 10.1016/j.yfrne.2008.02.001, PMID: 18374402 PMC2398769

[ref45] Burger H. G. (2002). Androgen production in women. Fertil. Steril. 77, 3–5. doi: 10.1016/S0015-0282(02)02985-0 12007895

[ref46] Burke S. M. Menks W. M. Cohen-Kettenis P. T. Klink D. T. Bakker J. (2014). Click-evoked Otoacoustic emissions in children and adolescents with gender identity disorder. Arch. Sex. Behav. 43, 1515–1523. doi: 10.1007/s10508-014-0278-2, PMID: 24567168

[ref47] Carter-Snell C. Hegadoren K. (2003). Stress disorders and gender: implications for theory and research. Can. J. Nurs. Res. 35, 34–55. PMID: 12908196

[ref48] Castberg I. Westin A. A. Skogvoll E. Spigset O. (2017). Effects of age and gender on the serum levels of clozapine, olanzapine, risperidone, and quetiapine. Acta Psychiatr. Scand. 136, 455–464. doi: 10.1111/acps.12794, PMID: 28865402

[ref49] Ceci S. J. Papierno P. B. (2005). The rhetoric and reality of gap closing: when the “have-nots” gain but the “haves” gain even more. Am. Psychol. 60:149:160. doi: 10.1037/0003-066X.60.2.149 15740447

[ref50] Ceylan-Isik A. F. McBride S. M. Ren J. (2010). Sex difference in alcoholism: who is at a greater risk for development of alcoholic complication? Life Sci. 87, 133–138. doi: 10.1016/j.lfs.2010.06.002, PMID: 20598716 PMC2913110

[ref51] Chai N. C. Peterlin B. L. Calhoun A. H. (2014). Migraine and estrogen. Curr. Opin. Neurol. 27, 315–324. doi: 10.1097/WCO.0000000000000091, PMID: 24792340 PMC4102139

[ref52] Chang S. C. Tucker T. Thorogood N. P. Brown C. J. (2006). Mechanisms of X-chromosome inactivation. Front. Biosci. 11, 852–866. doi: 10.2741/1842 16146776

[ref53] Chao A. M. Loughead J. Bakizada Z. M. Hopkins C. M. Geliebter A. Gur R. C.. (2017). Sex/gender differences in neural correlates of food stimuli: a systematic review of functional neuroimaging studies. Obes. Rev. 18, 687–699. doi: 10.1111/obr.12527, PMID: 28371180 PMC5549442

[ref54] Cisternas C. D. Garcia-Segura L. M. Cambiasso M. J. (2018). Hormonal and genetic factors interact to control aromatase expression in the developing brain. J. Neuroendocrinol. 30:535. doi: 10.1111/jne.12535, PMID: 28891264

[ref55] Clayton J. A. (2018). Applying the new SABV (sex as a biological variable) policy to research and clinical care. Physiol. Behav. 187, 2–5. doi: 10.1016/j.physbeh.2017.08.012, PMID: 28823546

[ref56] Cooke B. M. Woolley C. S. (2005). Sexually dimorphic synaptic organization of the medial amygdala. J. Neurosci. 25, 10759–10767. doi: 10.1523/JNEUROSCI.2919-05.2005, PMID: 16291949 PMC6725860

[ref57] Costa P. T. Terracciano A. McCrae R. R. (2001). Gender differences in personality traits across cultures: robust and surprising findings. J. Pers. Soc. Psychol. 81, 322–331. doi: 10.1037/0022-3514.81.2.322, PMID: 11519935

[ref58] Craft R. M. (2003). Sex differences in opioid analgesia: “from mouse to man”. Clin. J. Pain 19, 175–186. doi: 10.1097/00002508-200305000-00005 12792556

[ref59] Craft R. M. Stratmann J. A. Bartok R. E. Walpole T. I. King S. J. (1999). Sex differences in development of morphine tolerance and dependence in the rat. Psychopharmacology (Berl) 143, 1–7. doi: 10.1007/s002130050911 10227072

[ref60] Craig A. D. B. (2009). How do you feel–now? The anterior insula and human awareness. Nat. Rev. Neurosci. 10, 59–70. doi: 10.1038/nrn2555 19096369

[ref61] Cruz S. L. Rodríguez-Manzo G. (2000). Gender differences in the cardiovascular responses to morphine and naloxone in spinal rats. Eur. J. Pharmacol. 397, 121–128. doi: 10.1016/s0014-2999(00)00260-0 10844106

[ref62] Curtis A. L. Bethea T. Valentino R. J. (2006). Sexually dimorphic responses of the brain norepinephrine system to stress and Corticotropin-releasing factor. Neuropsychopharmacology 31, 544–554. doi: 10.1038/sj.npp.1300875, PMID: 16123744

[ref63] Czech D. P. Lee J. Sim H. Parish C. L. Vilain E. Harley V. R. (2012). The human testis-determining factor SRY localizes in midbrain dopamine neurons and regulates multiple components of catecholamine synthesis and metabolism. J. Neurochem. 122, 260–271. doi: 10.1111/j.1471-4159.2012.07782.x, PMID: 22568433 PMC3529967

[ref64] Dahan A. Sarton E. Teppema L. Olievier C. (1998). Sex-related differences in the influence of morphine on ventilatory control in humans. Anesthesiology 88, 903–913. doi: 10.1097/00000542-199804000-00009 9579498

[ref65] Dai Z. Ramesh V. Locasale J. W. (2020). The evolving metabolic landscape of chromatin biology and epigenetics. Nat. Rev. Genet. 21, 737–753. doi: 10.1038/s41576-020-0270-8, PMID: 32908249 PMC8059378

[ref66] Davatzikos C. Resnick S. M. (1998). Sex differences in anatomic measures of interhemispheric connectivity: correlations with cognition in women but not men. Cereb Cortex 8, 635–640. doi: 10.1093/cercor/8.7.635 9823484

[ref67] Davies S. J. C. Esler M. Nutt D. J. (2010). Anxiety - bridging the heart/mind divide. J. Psychopharmacol. 24, 633–638. doi: 10.1177/0269881109103800, PMID: 19346282

[ref68] Davies W. Isles A. R. Wilkinson L. S. (2001). Imprinted genes and mental dysfunction. Ann. Med. 33, 428–436. doi: 10.3109/07853890108995956 11585104

[ref69] De Rooij S. R. Caan M. W. A. Swaab D. F. Nederveen A. J. Majoie C. B. Schwab M.. (2016). Prenatal famine exposure has sex-specific effects on brain size. Brain 139, 2136–2142. doi: 10.1093/brain/aww132, PMID: 27401522

[ref70] Deakin J. F. W. (2003). Depression and antisocial personality disorder: two contrasting disorders of 5HT function. J. Neural Transm. Suppl. 64, 79–93. doi: 10.1007/978-3-7091-6020-6_5, PMID: 12830930

[ref71] Dessens A. B. Cohen-Kettenis P. T. Mellenbergh G. J. Poll N. V. D. Koppe J. G. Boer K. (1999). Prenatal exposure to anticonvulsants and psychosexual development. Arch. Sex. Behav. 28, 31–44. doi: 10.1023/A:1018789521375 10097803

[ref72] Devous M. D. Stokely E. M. Chehabi H. H. Bonte F. J. (1986). Normal distribution of regional cerebral blood flow measured by dynamic single-photon emission tomography. J. Cereb. Blood Flow Metab. 6, 95–104. doi: 10.1038/jcbfm.1986.12, PMID: 3484747

[ref73] Dewing P. Shi T. Horvath S. Vilain E. (2003). Sexually dimorphic gene expression in mouse brain precedes gonadal differentiation. Brain Res. Mol. Brain Res. 118, 82–90. doi: 10.1016/S0169-328X(03)00339-5, PMID: 14559357

[ref74] DeWulf V. Bottjer S. W. (2002). Age and sex differences in mitotic activity within the Zebra Finch telencephalon. J. Neurosci. 22, 4080–4094. doi: 10.1523/JNEUROSCI.22-10-04080.2002, PMID: 12019327 PMC6757632

[ref75] Domes G. Schulze L. Böttger M. Grossmann A. Hauenstein K. Wirtz P. H.. (2010). The neural correlates of sex differences in emotional reactivity and emotion regulation. Hum. Brain Mapp. 31, 758–769. doi: 10.1002/hbm.20903, PMID: 19957268 PMC6871188

[ref76] Dotto G. (2019). Gender and sex–time to bridge the gap. EMBO Mol. Med. 11:e10668. doi: 10.15252/emmm.201910668, PMID: 30979711 PMC6505576

[ref77] Elphick M. R. Egertová M. (2001). The neurobiology and evolution of cannabinoid signalling. Philos. Trans. R. Soc. Lond. Ser. B Biol. Sci. 356, 381–408. doi: 10.1098/rstb.2000.0787, PMID: 11316486 PMC1088434

[ref78] Else-Quest N. M. Hyde J. S. Linn M. C. (2010). Cross-national patterns of gender differences in mathematics: a meta-analysis. Psychol. Bull. 136, 103–127. doi: 10.1037/a0018053, PMID: 20063928

[ref79] Epperson C. N. Amin Z. Ruparel K. Gur R. Loughead J. (2012). Interactive effects of estrogen and serotonin on brain activation during working memory and affective processing in menopausal women. Psychoneuroendocrinology 37, 372–382. doi: 10.1016/j.psyneuen.2011.07.007, PMID: 21820247 PMC3226892

[ref142] Erdogan A. R. Dane S. Aydin M. D. Özdikici M. Diyarbakirli S. (2004). Sex and handedness differences in size of cerebral ventricles of normal subjects. Int. J. Neurosci. 114, 67–73. doi: 10.1080/00207450490249428, PMID: 14660068

[ref80] Ficek B. Horien C. Lacadie C. Shen X. Scheinost D. Constable T.. (2021). Sex differences in connectivity in the default mode network in healthy aging adults. Alzheimers Dement. 17:e056050. doi: 10.1002/alz.056050 PMC10183749 36563018

[ref81] Filipek P. A. Richelme C. Kennedy D. N. Caviness V. S. (1994). The young adult human brain: an MRI-based morphometric analysis. Cereb. Cortex 4, 344–360. doi: 10.1093/cercor/4.4.344, PMID: 7950308

[ref82] Flerkó B. (1971). Steroid hormones and the differentiation of the central nervous system. Curr. Top. Exp. Endocrinol. 1, 41–80. doi: 10.1016/B978-0-12-153201-7.50008-9 4350827

[ref83] Foreman M. Hare L. York K. Balakrishnan K. Sánchez F. J. Harte F.. (2019). Genetic link between gender dysphoria and sex hormone signaling. J. Clin. Endocrinol. Metab. 104, 390–396. doi: 10.1210/jc.2018-01105, PMID: 30247609

[ref84] Freeman R. D. Fast D. K. Burd L. Kerbeshian J. Robertson M. M. Sandor P. (2000). An international perspective on Tourette syndrome: selected findings from 3,500 individuals in 22 countries. Dev. Med. Child Neurol. 42, 436–447. doi: 10.1111/j.1469-8749.2000.tb00346.x, PMID: 10972415

[ref85] Galanopoulou A. S. Alm E. M. Velísková J. (2003). Estradiol reduces seizure-induced hippocampal injury in ovariectomized female but not in male rats. Neurosci. Lett. 342, 201–205. doi: 10.1016/S0304-3940(03)00282-9, PMID: 12757899

[ref86] Gamazon E. R. Stranger B. E. (2014). Genomics of alternative splicing: evolution, development and pathophysiology. Hum. Genet. 133, 679–687. doi: 10.1007/s00439-013-1411-3, PMID: 24378600

[ref87] Garant D. S. Gale K. (1983). Lesions of substantia nigra protect against experimentally induced seizures. Brain Res. 273:156:161. doi: 10.1016/0006-8993(83)91105-8 6616222

[ref88] Garden G. M. F. Rothery D. J. (1992). A female monozygotic twin pair discordant for transsexualism: some theoretical implications. Br. J. Psychiatry 161, 852–854. doi: 10.1192/bjp.161.6.852, PMID: 1483176

[ref89] Geary D. (1998). Male, female: the evolution of human sex differences. Washington, DC: American Psychological Association.

[ref90] Gennatas E. D. Avants B. B. Wolf D. H. Satterthwaite T. D. Ruparel K. Ciric R.. (2017). Age-related effects and sex differences in gray matter density, volume, mass, and cortical thickness from childhood to Young adulthood. J. Neurosci. 37, 5065–5073. doi: 10.1523/JNEUROSCI.3550-16.2017, PMID: 28432144 PMC5444192

[ref91] Gentner M. B. Leppert M. L. O. (2019). Environmental influences on health and development: nutrition, substance exposure, and adverse childhood experiences. Dev. Med. Child Neurol. 61, 1008–1014. doi: 10.1111/dmcn.14149, PMID: 30671935

[ref92] Georgiev D. Hamberg K. Hariz M. Forsgren L. Hariz G. M. (2017). Gender differences in Parkinson’s disease: a clinical perspective. Acta Neurol. Scand. 136, 570–584. doi: 10.1111/ane.12796, PMID: 28670681

[ref93] Gershon J. (2002). A meta-analytic review of gender differences in ADHD. J. Atten. Disord. 5, 143–154. doi: 10.1177/108705470200500302, PMID: 11911007

[ref94] Ghahramani N. M. Ngun T. C. Chen P. Y. Tian Y. Krishnan S. Muir S.. (2014). The effects of perinatal testosterone exposure on the DNA methylome of the mouse brain are late-emerging. Biol. Sex Differ. 5:8. doi: 10.1186/2042-6410-5-8, PMID: 24976947 PMC4074311

[ref95] Giacobini E. Pepeu G. (2018). Sex and gender differences in the brain cholinergic system and in the response to therapy of Alzheimer disease with cholinesterase inhibitors. Curr. Alzheimer Res. 15, 1077–1084. doi: 10.2174/1567205015666180613111504, PMID: 29895246

[ref96] Giedd J. N. Castellanos F. X. Rajapakse J. C. Vaituzis A. C. Rapoport J. L. (1997). Sexual dimorphism of the developing human brain. Prog. Neuro-Psychopharmacol. Biol. Psychiatry 21, 1185–1201. doi: 10.1016/S0278-5846(97)00158-9 9460086

[ref97] Giedd J. N. Raznahan A. Mills K. L. Lenroot R. K. (2012). Review: magnetic resonance imaging of male/female differences in human adolescent brain anatomy. Biol. Sex Differ. 3:19. doi: 10.1186/2042-6410-3-19, PMID: 22908911 PMC3472204

[ref98] Gingnell M. Bannbers E. Wikström J. Fredrikson M. Sundström-Poromaa I. (2013). Premenstrual dysphoric disorder and prefrontal reactivity during anticipation of emotional stimuli. Eur. Neuropsychopharmacol. 23, 1474–1483. doi: 10.1016/j.euroneuro.2013.08.002, PMID: 24001875

[ref99] Glickman S. E. Short R. V. Renfree M. B. (2005). Sexual differentiation in three unconventional mammals: spotted hyenas, elephants and tammar wallabies. Horm. Behav. 48, 403–417. doi: 10.1016/j.yhbeh.2005.07.013, PMID: 16197946

[ref100] Gobinath A. R. Choleris E. Galea L. A. M. (2017). Sex, hormones, and genotype interact to influence psychiatric disease, treatment, and behavioral research. J. Neurosci. Res. 95, 50–64. doi: 10.1002/jnr.23872, PMID: 27870452

[ref101] Goetz T. Bieg M. Lüdtke O. Pekrun R. Hall N. C. (2013). Do girls really experience more anxiety in mathematics? Psychol. Sci. 24, 2079–2087. doi: 10.1177/0956797613486989 23985576

[ref102] Goldstein J. M. Seidman L. J. Horton N. J. Makris N. Kennedy D. N. Caviness V. S.. (2001). Normal sexual dimorphism of the adult human brain assessed by in vivo magnetic resonance imaging. Cereb. Cortex 11, 490–497. doi: 10.1093/cercor/11.6.490, PMID: 11375910

[ref103] Gomez-Santos C. Hernandez-Morante J. J. Margareto J. Larrarte E. Formiguera X. Martínez C. M.. (2011). Profile of adipose tissue gene expression in premenopausal and postmenopausal women: site-specific differences. Menopause 18, 675–684. doi: 10.1097/gme.0b013e31820641da, PMID: 21358552

[ref104] Gou Z. H. Wang X. Wang W. (2012). Evolution of neurotransmitter gamma-aminobutyric acid, glutamate and their receptors. Dongwuxue Yanjiu 33, E75–E81. doi: 10.3724/SP.J.1141.2012.E05-06E75, PMID: 23266985

[ref105] Green R. (2000). Family Cooccurrence of “gender dysphoria”: ten sibling or parent–child pairs. Arch. Sex. Behav. 29, 499–507. doi: 10.1023/A:1001947920872 10983252

[ref106] Grimm S. L. Hartig S. M. Edwards D. P. (2016). Progesterone receptor signaling mechanisms. J. Mol. Biol. 428, 3831–3849. doi: 10.1016/j.jmb.2016.06.020, PMID: 27380738

[ref107] Guglielmi V. Vulink N. C. C. Denys D. Wang Y. Samuels J. F. Nestadt G. (2014). Obsessive-compulsive disorder and female reproductive cycle events: results from the OCD and reproduction collaborative study. Depress. Anxiety 31, 979–987. doi: 10.1002/da.22234, PMID: 24421066

[ref108] Guiso L. Monte F. Sapienza P. Zingales L. (2008). DIVERSITY: Culture, Gender, and Math. Science 320, 1164–1165. doi: 10.1126/science.1154094 18511674

[ref900] Guo S. Zhou Y. Zeng P. Xu G. Wang G. Cui O. (2016). Identification and analysis of the human sex-biased genes. Brief. Bioinformat. 19, 188–198. doi: 10.1093/bib/bbw125, PMID: 28028006

[ref109] Gur R. E. Gur R. C. (1990). Gender differences in regional cerebral blood flow. Schizophr. Bull. 16, 247–254. doi: 10.1093/schbul/16.2.247 2374883

[ref110] Gur R. C. Gur R. E. (2017). Complementarity of sex differences in brain and behavior: from laterality to multimodal neuroimaging. J. Neurosci. Res. 95, 189–199. doi: 10.1002/jnr.23830, PMID: 27870413 PMC5129843

[ref111] Gur R. C. Richard J. Calkins M. E. Chiavacci R. Hansen J. A. Bilker W. B.. (2012). Age group and sex differences in performance on a computerized neurocognitive battery in children age 8-21. Neuropsychology 26, 251–265. doi: 10.1037/a0026712, PMID: 22251308 PMC3295891

[ref112] Gur R. C. Turetsky B. I. Matsui M. Yan M. Bilker W. Hughett P.. (1999). Sex differences in brain gray and white matter in healthy young adults: correlations with cognitive performance. J. Neurosci. 19, 4065–4072. doi: 10.1523/JNEUROSCI.19-10-04065.1999, PMID: 10234034 PMC6782697

[ref113] Haast R. A. M. Gustafson D. R. Kiliaan A. J. (2012). Sex differences in stroke. J. Cereb. Blood Flow Metab. 32, 2100–2107. doi: 10.1038/jcbfm.2012.141, PMID: 23032484 PMC3519418

[ref114] Hahn A. Kranz G. S. Küblböck M. Kaufmann U. Ganger S. Hummer A.. (2015). Structural connectivity networks of transgender people. Cereb. Cortex 25, 3527–3534. doi: 10.1093/cercor/bhu194, PMID: 25217469 PMC4585501

[ref115] Haier R. J. Jung R. E. Yeo R. A. Head K. Alkire M. T. (2005). The neuroanatomy of general intelligence: sex matters. Neuroimage 25, 320–327. doi: 10.1016/j.neuroimage.2004.11.019, PMID: 15734366

[ref116] Hall E. Volkov P. Dayeh T. Esguerra J. L. S. Salö S. Eliasson L.. (2014). Sex differences in the genome-wide DNA methylation pattern and impact on gene expression, microRNA levels and insulin secretion in human pancreatic islets. Genome Biol. 15:522. doi: 10.1186/s13059-014-0522-z, PMID: 25517766 PMC4256841

[ref117] Halpern D. F. (2021). Sex Differences in Cognitive Abilities. Abingdon: Routledge, CRC Press.

[ref118] Halpern D. F. Benbow C. P. Geary D. C. Gur R. C. Hyde J. S. Gernsbacher M. A. (2007). The science of sex differences in science and mathematics. Psychol. Sci. Public Interest 8, 1–51. doi: 10.1111/j.1529-1006.2007.00032.x, PMID: 25530726 PMC4270278

[ref119] Halpern D. F. LaMay M. L. (2000). The smarter sex: a critical review of sex differences in intelligence. Educ. Psychol. Rev. 12, 229–246. doi: 10.1023/A:1009027516424

[ref120] Hampson E. (1990). Estrogen-related variations in human spatial and articulatory-motor skills. Psychoneuroendocrinology 15, 97–111. doi: 10.1016/0306-4530(90)90018-5 2359813

[ref121] Harasty J. Double K. L. Halliday G. M. Kril J. J. McRitchie D. A. (1997). Language-associated cortical regions are proportionally larger in the female brain. Arch. Neurol. 54, 171–176. doi: 10.1001/archneur.1997.00550140045011, PMID: 9041858

[ref122] Hatazawa J. Brooks R. A. Di Chiro G. Campbell G. (1987). Global cerebral glucose utilization is independent of brain size: a PET study. J. Comput. Assist. Tomogr. 11, 571–576. doi: 10.1097/00004728-198707000-00002, PMID: 3496367

[ref123] Hatcher R. Hatcher S. Berlin M. Okla K. Richards J. (1990). Psychological mindedness and abstract reasoning in late childhood and adolescence: an exploration using new instruments. J. Youth Adolesc. 19, 307–326. doi: 10.1007/BF01537075, PMID: 24272530

[ref124] Hausmann M. Güntürkün O. (2000). Steroid fluctuations modify functional cerebral asymmetries: the hypothesis of progesterone-mediated interhemispheric decoupling. Neuropsychologia 38, 1362–1374. doi: 10.1016/S0028-3932(00)00045-2, PMID: 10869579

[ref125] Hayward C. Sanborn K. (2002). Puberty and the emergence of gender differences in psychopathology. J. Adolesc. Health 30, 49–58. doi: 10.1016/S1054-139X(02)00336-1, PMID: 11943575

[ref126] Hedden T. Ketay S. Aron A. Markus H. R. Gabrieli J. D. E. (2008). Cultural influences on neural substrates of attentional control. Psychol. Sci. 19, 12–17. doi: 10.1111/j.1467-9280.2008.02038.x, PMID: 18181784

[ref127] Heidari S. Babor T. F. Castro P. D. Tort S. Curno M. (2017). Sex and gender equity in research: rationale for the SAGER guidelines and recommended use. Epidemiol. Serv. Saude 26, 665–676. doi: 10.5123/S1679-49742017000300025, PMID: 28443945

[ref128] Heylens G. De Cuypere G. Zucker K. J. Schelfaut C. Elaut E. Vanden Bossche H.. (2012). Gender identity disorder in twins: a review of the case report literature. J. Sex. Med. 9, 751–757. doi: 10.1111/j.1743-6109.2011.02567.x, PMID: 22146048

[ref129] Hines M. Pasterski V. Spencer D. Neufeld S. Patalay P. Hindmarsh P. C.. (2016). Prenatal androgen exposure alters girls’ responses to information indicating gender-appropriate behaviour. Philos. Trans. R. Soc. B Biol. Sci. 371:20150125. doi: 10.1098/rstb.2015.0125, PMID: 26833843 PMC4785908

[ref130] Ho A. J. Raji C. A. Becker J. T. Lopez O. L. Kuller L. H. Hua X.. (2010). Obesity is linked with lower brain volume in 700 AD and MCI patients. Neurobiol. Aging 31, 1326–1339. doi: 10.1016/j.neurobiolaging.2010.04.006, PMID: 20570405 PMC3197833

[ref131] Hoekzema E. Schagen S. E. E. Kreukels B. P. C. Veltman D. J. Cohen-Kettenis P. T. Delemarre-van de Waal H.. (2015). Regional volumes and spatial volumetric distribution of gray matter in the gender dysphoric brain. Psychoneuroendocrinology 55, 59–71. doi: 10.1016/j.psyneuen.2015.01.016, PMID: 25720349

[ref132] Hoffman M. Gneezy U. List J. A. (2011). Nurture affects gender differences in spatial abilities. Proc. Natl. Acad. Sci. 108, 14786–14788. doi: 10.1073/pnas.1015182108, PMID: 21876159 PMC3169128

[ref133] Horie I. Abiru N. Eto M. Sako A. Akeshima J. Nakao T.. (2018). Sex differences in insulin and glucagon responses for glucose homeostasis in young healthy Japanese adults. J. Diabetes Investig. 9, 1283–1287. doi: 10.1111/jdi.12829, PMID: 29489067 PMC6215950

[ref134] Horstink M. W. I. M. Strijks E. Dluzen D. E. (2003). Estrogen and Parkinson’s disease. Adv. Neurol. 91, 107–114. PMID: 12442669

[ref135] Hoyle C. H. V. (2011). Evolution of neuronal signalling: transmitters and receptors. Auton. Neurosci. 165, 28–53. doi: 10.1016/j.autneu.2010.05.007, PMID: 20646967

[ref136] Hu H. Real E. Takamiya K. Kang M. G. Ledoux J. Huganir R. L.. (2007). Emotion enhances learning via norepinephrine regulation of AMPA-receptor trafficking. Cell 131, 160–173. doi: 10.1016/j.cell.2007.09.017, PMID: 17923095

[ref137] Hull L. Petrides K. V. Mandy W. (2020). The female autism phenotype and camouflaging: a narrative review. Rev. J. Autism. Dev. Disord. 7, 306–317. doi: 10.1007/s40489-020-00197-9

[ref138] Hyde C. Kenna J. C. (1977). A male M Z twin pair, concordant for transsexualism, discordant for schizophrenia. Acta Psychiatr. Scand. 56, 265–273. doi: 10.1111/j.1600-0447.1977.tb00227.x, PMID: 562589

[ref139] Iliescu D. Ilie A. Ispas D. Dobrean A. Clinciu A. I. (2016). Sex differences in intelligence: a multi-measure approach using nationally representative samples from Romania. Intelligence 58, 54–61. doi: 10.1016/j.intell.2016.06.007

[ref140] Im K. Lee J. M. Lee J. Shin Y. W. Kim I. Y. Kwon J. S.. (2006). Gender difference analysis of cortical thickness in healthy young adults with surface-based methods. NeuroImage 31, 31–38. doi: 10.1016/j.neuroimage.2005.11.042, PMID: 16426865

[ref141] Ingalhalikar M. Smith A. Parker D. Satterthwaite T. D. Elliott M. A. Ruparel K.. (2014). Sex differences in the structural connectome of the human brain. Proc. Natl. Acad. Sci. USA 111, 823–828. doi: 10.1073/pnas.1316909110, PMID: 24297904 PMC3896179

[ref143] Isensee J. Ruiz N. P. (2007). Sexually dimorphic gene expression in mammalian somatic tissue. Gend. Med. 4, S75–S95. doi: 10.1016/S1550-8579(07)80049-0 18156105

[ref144] Jaenisch R. Bird A. (2003). Epigenetic regulation of gene expression: how the genome integrates intrinsic and environmental signals. Nat. Genet. 33, 245–254. doi: 10.1038/ng1089, PMID: 12610534

[ref145] Jäncke L. (2009). The plastic human brain. Restor. Neurol. Neurosci. 27, 521–538. doi: 10.3233/RNN-2009-0519, PMID: 19847074

[ref146] Jäncke L. Mérillat S. Liem F. Hänggi J. (2015). Brain size, sex, and the aging brain. Hum. Brain Mapp. 36, 150–169. doi: 10.1002/hbm.22619, PMID: 25161056 PMC6869393

[ref147] Joel D. Berman Z. Tavor I. Wexler N. Gaber O. Stein Y.. (2015). Sex beyond the genitalia: the human brain mosaic. Proc. Natl. Acad. Sci. 112, 15468–15473. doi: 10.1073/pnas.1509654112, PMID: 26621705 PMC4687544

[ref148] Jones K. Johnson K. A. Becker J. A. Spiers P. A. Albert M. S. Holman B. L. (1998). Use of singular value decomposition to characterize age and gender differences in SPECT cerebral perfusion. J. Nucl. Med. 39, 965–973. PMID: 9627327

[ref149] Jost A. (1978). Basic sexual trends in the development of vertebrates. Ciba Found. Symp. 62, 5–18. doi: 10.1002/9780470720448.ch2 256834

[ref150] Juraska J. M. Sisk C. L. DonCarlos L. L. (2013). Sexual differentiation of the adolescent rodent brain: hormonal influences and developmental mechanisms. Horm. Behav. 64, 203–210. doi: 10.1016/j.yhbeh.2013.05.010, PMID: 23998664

[ref151] Kaasinen V. Någren K. Hietala J. Farde L. Rinne J. O. (2001). Sex differences in extrastriatal dopamine d2-like receptors in the human brain. Am J Psychiatry 158, 308–311. doi: 10.1176/appi.ajp.158.2.308 11156817

[ref152] Kalkbrenner K. A. Standley C. A. (2003). Estrogen modulation of NMDA-induced seizures in ovariectomized and non-ovariectomized rats. Brain Res. 964, 244–249. doi: 10.1016/S0006-8993(02)04065-9, PMID: 12576185

[ref901] Kassam I. Wu Y. Yang J. Visscher P. M. McRae A. F. (2019). Tissue-specific sex differences in human gene expression. Hum. Mol. Gen. 28, 2976–2986. doi: 10.1093/hmg/ddz090 31044242 PMC6736104

[ref153] Kasson B. G. George R. (1984). Endocrine influences on the actions of morphine: IV. Effects of sex and strain. Life Sci. 34, 1627–1634. 6727540 10.1016/0024-3205(84)90633-7

[ref154] Kauko A. Aittokallio J. Vaura F. Gen F. Ji H. Ebinger J. E.. (2021). Sex differences in genetic risk for hypertension. Hypertension 78, 1153–1155. doi: 10.1161/HYPERTENSIONAHA.121.17796, PMID: 34397277 PMC8429138

[ref155] Kempermann G. Gast D. Gage F. H. (2002). Neuroplasticity in old age: Sustained fivefold induction of hippocampal neurogenesis by long-term environmental enrichment. Ann. Neurol. 52, 135–143. doi: 10.1002/ana.10262 12210782

[ref156] Kennedy D. P. Adolphs R. (2012). The social brain in psychiatric and neurological disorders. Trends Cogn. Sci. 16, 559–572. doi: 10.1016/j.tics.2012.09.006 23047070 PMC3606817

[ref157] Kest B. Adler M. Hopkins E. (2000). Sex differences in thermoregulation after acute and chronic morphine administration in mice. Neurosci. Lett. 291, 126–128. doi: 10.1016/s0304-3940(00)01393-8 10978590

[ref158] Kharabian Masouleh S. Beyer F. Lampe L. Loeffler M. Luck T. Riedel-Heller S. G.. (2018). Gray matter structural networks are associated with cardiovascular risk factors in healthy older adults. J. Cereb. Blood Flow Metab. 38, 360–372. doi: 10.1177/0271678X17729111, PMID: 28857651 PMC5951018

[ref159] Kilts C. D. Gross R. E. Ely T. D. Drexler K. P. G. (2004). The neural correlates of cue-induced craving in cocaine-dependent women. Am. J. Psychiatry 161, 233–241. doi: 10.1176/appi.ajp.161.2.233, PMID: 14754771

[ref160] Kimura D. (1999). Sex and cognition. Cambridge, MA: MIT Press, p. 234.

[ref161] Klein L. C. Corwin E. J. (2002). Seeing the unexpected: how sex differences in stress responses may provide a new perspective on the manifestation of psychiatric disorders. Curr. Psychiatry Rep. 4, 441–448. doi: 10.1007/s11920-002-0072-z, PMID: 12441024

[ref162] Klein S. L. Flanagan K. L. (2016). Sex differences in immune responses. Nat. Rev. Immunol. 16, 626–638. doi: 10.1038/nri.2016.90 27546235

[ref163] Knickmeyer R. C. Wang J. Zhu H. Geng X. Woolson S. Hamer R. M.. (2014). Impact of sex and gonadal steroids on neonatal brain structure. Cereb. Cortex 24, 2721–2731. doi: 10.1093/cercor/bht125, PMID: 23689636 PMC4153808

[ref164] Kofink D. Boks M. P. M. Timmers H. T. M. Kas M. J. (2013). Epigenetic dynamics in psychiatric disorders: environmental programming of neurodevelopmental processes. Neurosci. Biobehav. Rev. 37, 831–845. doi: 10.1016/j.neubiorev.2013.03.020, PMID: 23567520

[ref165] Koldzic-Zivanovic N. Seitz P. K. Watson C. S. Cunningham K. A. Thomas M. L. (2004). Intracellular signaling involved in estrogen regulation of serotonin reuptake. Mol. Cell. Endocrinol. 226, 33–42. doi: 10.1016/j.mce.2004.07.017, PMID: 15489003

[ref166] Kósa J. P. Balla B. Speer G. Kiss J. Borsy A. Podani J.. (2009). Effect of menopause on gene expression pattern in bone tissue of nonosteoporotic women. Menopause 16, 367–377. doi: 10.1097/gme.0b013e318188b260, PMID: 19512969

[ref167] Kotsopoulos I. A. W. van Merode T. Kessels F. G. H. de Krom M. C. T. F. M. Knottnerus J. A. (2002). Systematic review and meta-analysis of incidence studies of epilepsy and unprovoked seizures. Epilepsia 43, 1402–1409. doi: 10.1046/j.1528-1157.2002.t01-1-26901.x, PMID: 12423392

[ref168] Kranz G. S. Hahn A. Kaufmann U. Küblböck M. Hummer A. Ganger S.. (2014). White matter microstructure in transsexuals and controls investigated by diffusion tensor imaging. J. Neurosci. 34, 15466–15475. doi: 10.1523/JNEUROSCI.2488-14.2014, PMID: 25392513 PMC4699258

[ref169] Kudwa A. E. Bodo C. Gustafsson J. A. Rissman E. F. (2005). A previously uncharacterized role for estrogen receptor beta: defeminization of male brain and behavior. Proc. Natl. Acad. Sci. USA 102, 4608–4612. doi: 10.1073/pnas.0500752102, PMID: 15761056 PMC555526

[ref170] Kuehner C. (2003). Gender differences in unipolar depression: an update of epidemiological findings and possible explanations. Acta Psychiatr. Scand. 108, 163–174. doi: 10.1034/j.1600-0447.2003.00204.x 12890270

[ref171] Laakso A. Vilkman H. Bergman J. Haaparanta M. Solin O. Syvälahti E.. (2002). Sex differences in striatal presynaptic dopamine synthesis capacity in healthy subjects. Biol. Psychiatry 52, 759–763. doi: 10.1016/S0006-3223(02)01369-0, PMID: 12372667

[ref172] Labonté B. Engmann O. Purushothaman I. Menard C. Wang J. Tan C.. (2017). Sex-specific transcriptional signatures in human depression. Nat. Med. 23, 1102–1111. doi: 10.1038/nm.4386, PMID: 28825715 PMC5734943

[ref173] Lakin J. M. (2013). Sex differences in reasoning abilities: surprising evidence that male–female ratios in the tails of the quantitative reasoning distribution have increased. Intelligence 41, 263–274. doi: 10.1016/j.intell.2013.04.004

[ref174] Leitão E. Schröder C. Parenti I. Dalle C. Rastetter A. Kühnel T.. (2022). Systematic analysis and prediction of genes associated with monogenic disorders on human chromosome X. Nat. Commun. 13:264. doi: 10.1038/s41467-022-34264-y 36323681 PMC9630267

[ref175] Leslie E. Wilson R. (2020). Sheltering in place and domestic violence: evidence from calls for service during COVID-19. J. Public Econ. 189:104241. doi: 10.1016/j.jpubeco.2020.104241, PMID: 32834179 PMC7377795

[ref176] Levine S. C. Foley A. Lourenco S. Ehrlich S. Ratliff K. (2016). Sex differences in spatial cognition: advancing the conversation. WIREs Cogn. Sci. 7, 127–155. doi: 10.1002/wcs.1380, PMID: 26825049

[ref177] Lindberg S. M. Hyde J. S. Petersen J. L. Linn M. C. (2010). New trends in gender and mathematics performance: a meta-analysis. Psychol. Bull. 136, 1123–1135. doi: 10.1037/a0021276, PMID: 21038941 PMC3057475

[ref178] Lindholm M. E. Huss M. Solnestam B. W. Kjellqvist S. Lundeberg J. Sundberg C. J. (2014). The human skeletal muscle transcriptome: sex differences, alternative splicing, and tissue homogeneity assessed with RNA sequencing. FASEB J. 28, 4571–4581. doi: 10.1096/fj.14-255000, PMID: 25016029

[ref179] Liu F. Day M. Muñiz L. C. Bitran D. Arias R. Revilla-Sanchez R.. (2008). Activation of estrogen receptor-beta regulates hippocampal synaptic plasticity and improves memory. Nat. Neurosci. 11, 334–343. doi: 10.1038/nn2057, PMID: 18297067

[ref180] Lombardo M. V. Ashwin E. Auyeung B. Chakrabarti B. Taylor K. Hackett G.. (2012). Fetal testosterone influences sexually dimorphic gray matter in the human brain. J. Neurosci. 32, 674–680. doi: 10.1523/JNEUROSCI.4389-11.2012, PMID: 22238103 PMC3306238

[ref181] Löscher W. Czuczwar S. J. Jäckel R. Schwarz M. (1987). Effect of microinjections of gamma-vinyl GABA or isoniazid into substantia nigra on the development of amygdala kindling in rats. Exp. Neurol. 95, 622–638. doi: 10.1016/0014-4886(87)90304-9 3817084

[ref182] Luders E. Narr K. L. Thompson P. M. Rex D. E. Jancke L. Steinmetz H.. (2004). Gender differences in cortical complexity. Nat. Neurosci. 7, 799–800. doi: 10.1038/nn1277 15338563

[ref183] Luders E. Narr K. L. Thompson P. M. Rex D. E. Woods R. P. Deluca H.. (2006a). Gender effects on cortical thickness and the influence of scaling. Hum. Brain Mapp. 27, 314–324. doi: 10.1002/hbm.20187, PMID: 16124013 PMC6871390

[ref184] Luders E. Thompson P. M. Narr K. L. Toga A. W. Jancke L. Gaser C. (2006b). A curvature-based approach to estimate local gyrification on the cortical surface. NeuroImage 29, 1224–1230. doi: 10.1016/j.neuroimage.2005.08.049, PMID: 16223589

[ref185] Lui K. F. Yip K. H. Wong A. C. N. (2021). Gender differences in multitasking experience and performance. Q. J. Exp. Psychol. 74, 344–362. doi: 10.1177/1747021820960707, PMID: 32933422

[ref186] Lupien S. J. McEwen B. S. Gunnar M. R. Heim C. (2009). Effects of stress throughout the lifespan on the brain, behaviour and cognition. Nat. Rev. Neurosci. 10, 434–445. doi: 10.1038/nrn2639 19401723

[ref187] Lynch W. J. Roth M. E. Carroll M. E. (2002). Biological basis of sex differences in drug abuse: preclinical and clinical studies. Psychopharmacology 164, 121–137. doi: 10.1007/s00213-002-1183-2, PMID: 12404074

[ref188] Ma J. Malladi S. Beck A. H. (2016). Systematic analysis of sex-linked molecular alterations and therapies in Cancer. Sci. Rep. 6:19119. doi: 10.1038/srep19119, PMID: 26755347 PMC4709570

[ref189] Maeda Y. Yoon S. Y. (2013). A Meta-analysis on gender differences in mental rotation ability measured by the Purdue spatial visualization tests: visualization of rotations (PSVT:R). Educ. Psychol. Rev. 25, 69–94. doi: 10.1007/s10648-012-9215-x

[ref190] Maggi R. Limonta P. Dondi D. Piva F. (1991). Modulation of the binding characteristics of hypothalamic mu opioid receptors in rats by gonadal steroids. J. Steroid Biochem. Mol. Biol. 40, 113–121. doi: 10.1016/0960-0760(91)90174-4 1659872

[ref191] Mank J. E. Rideout E. J. (2021). Developmental mechanisms of sex differences: from cells to organisms. Development 148:dev199750. doi: 10.1242/dev.199750 34647574

[ref192] Manning K. S. Cooper T. A. (2017). The roles of RNA processing in translating genotype to phenotype. Nat. Rev. Mol. Cell Biol. 18, 102–114. doi: 10.1038/nrm.2016.139, PMID: 27847391 PMC5544131

[ref193] Marino M. Galluzzo P. Ascenzi P. (2006). Estrogen signaling multiple pathways to impact gene transcription. Curr. Genomics 7, 497–508. doi: 10.2174/138920206779315737, PMID: 18369406 PMC2269003

[ref194] Marrazzi M. A. McQuarters A. Barnes C. Lawhorn J. D’Amico-Rasmussen Q. (1996). Male/female comparison of morphine effect on food intake--relation to anorexia nervosa. Pharmacol. Biochem. Behav 53, 433–435. doi: 10.1016/0091-3057(95)02013-6 8808154

[ref195] Marwha D. Halari M. Eliot L. (2017). Meta-analysis reveals a lack of sexual dimorphism in human amygdala volume. NeuroImage 147, 282–294. doi: 10.1016/j.neuroimage.2016.12.021, PMID: 27956206

[ref196] May A. (2011). Experience-dependent structural plasticity in the adult human brain. Trends Cogn. Sci. 15, 475–482. doi: 10.1016/j.tics.2011.08.002 21906988

[ref197] Mazure C. M. Swendsen J. (2016). Sex differences in Alzheimer’s disease and other dementias. Lancet Neurol. 15, 451–452. doi: 10.1016/S1474-4422(16)00067-3, PMID: 26987699 PMC4864429

[ref198] McCarthy M. M. Arnold A. P. (2011). Reframing sexual differentiation of the brain. Nat. Neurosci. 14, 677–683. doi: 10.1038/nn.2834, PMID: 21613996 PMC3165173

[ref199] McEwen B. S. (1972). Steroid hormones and the chemistry of behavior. Adv. Behav. Biol. 4, 41–59. doi: 10.1007/978-1-4684-3060-8_3 4376092

[ref200] McEwen B. S. (2001). Invited Review: Estrogens effects on the brain: multiple sites and molecular mechanisms. J. Appl. Physiol. 91, 2785–2801. doi: 10.1152/jappl.2001.91.6.2785 11717247

[ref201] McEwen B. S. (2007). Physiology and neurobiology of stress and adaptation: central role of the brain. Physiol. Rev. 87, 873–904. doi: 10.1152/physrev.00041.2006, PMID: 17615391

[ref202] McEwen B. S. Milner T. A. (2017). Understanding the broad influence of sex hormones and sex differences in the brain. J. Neurosci. Res. 95, 24–39. doi: 10.1002/jnr.23809, PMID: 27870427 PMC5120618

[ref203] McFadden D. Pasanen E. G. (1998). Comparison of the auditory systems of heterosexuals and homosexuals: click-evoked otoacoustic emissions. Proc. Natl. Acad. Sci. 95, 2709–2713. doi: 10.1073/pnas.95.5.2709, PMID: 9482952 PMC19471

[ref204] Mennecozzi M. Landesmann B. Palosaari T. Harris G. Whelan M. (2015). Sex differences in liver toxicity-do female and male human primary hepatocytes react differently to toxicants in vitro? PLoS One 10:e0122786. doi: 10.1371/journal.pone.0122786, PMID: 25849576 PMC4388670

[ref205] Migeon B. R. (2007). Why females are mosaics, X-chromosome inactivation, and sex differences in disease. Gend. Med. 4, 97–105. doi: 10.1016/S1550-8579(07)80024-6, PMID: 17707844

[ref206] Minagar A. Sevush S. Bertran A. (2000). Cerebral ventricles are smaller in Hispanic than non-Hispanic patients with Alzheimer’s disease. Neurology 55, 446–448. doi: 10.1212/WNL.55.3.446 10932287

[ref207] Moen J. K. Lee A. M. (2021). Sex differences in the nicotinic acetylcholine receptor system of rodents: impacts on nicotine and alcohol reward behaviors. Front. Neurosci. 15:745783. doi: 10.3389/fnins.2021.745783, PMID: 34621155 PMC8490611

[ref208] Möller-Leimkühler A. M. (2002). Barriers to help-seeking by men: a review of sociocultural and clinical literature with particular reference to depression. J. Affect. Disord. 71, 1–9. doi: 10.1016/S0165-0327(01)00379-2, PMID: 12167495

[ref209] Moncrieff J. Cooper R. E. Stockmann T. Amendola S. Hengartner M. P. Horowitz M. A. (2022). The serotonin theory of depression: a systematic umbrella review of the evidence. Mol. Psychiatry 28, 3243–3256. doi: 10.1038/s41380-022-01661-0 35854107 PMC10618090

[ref210] Mozley L. H. Gur R. C. Mozley P. D. Gur R. E. (2001). Striatal dopamine transporters and cognitive functioning in healthy men and women. Am. J. Psychiatry 158, 1492–1499. doi: 10.1176/appi.ajp.158.9.1492 11532737

[ref211] Mungas D. Reed B. R. Farias S. T. DeCarli C. (2009). Age and education effects on relationships of cognitive test scores with brain structure in demographically diverse older persons. Psychol. Aging 24, 116–128. doi: 10.1037/a0013421, PMID: 19290743 PMC2861868

[ref212] Munkvold L. H. Lundervold A. J. Manger T. (2011). Oppositional defiant disorder-gender differences in co-occurring symptoms of mental health problems in a general population of children. J. Abnorm. Child Psychol. 39, 577–587. doi: 10.1007/s10802-011-9486-6, PMID: 21243524

[ref213] Münte T. F. Altenmüller E. Jäncke L. (2002). The musician’s brain as a model of neuroplasticity. Nat. Rev. Neurosci. 3, 473–478. doi: 10.1038/nrn843, PMID: 12042882

[ref214] Murphy V. E. Gibson P. G. (2008). Premenstrual asthma: prevalence, cycle-to-cycle variability and relationship to oral contraceptive use and menstrual symptoms. J. Asthma 45, 696–704. doi: 10.1080/02770900802207279, PMID: 18951263

[ref215] Neisser U. Boodoo G. Bouchard T. J. Jr. Boykin A. W. Brody N. Ceci S. J.. (1996). Intelligence: knowns and unknowns. Am. Psychol. 51, 77–101. doi: 10.1037/0003-066X.51.2.77

[ref216] Neufang S. Specht K. Hausmann M. Güntürkün O. Herpertz-Dahlmann B. Fink G. R.. (2009). Sex differences and the impact of steroid hormones on the developing human brain. Cereb. Cortex 19, 464–473. doi: 10.1093/cercor/bhn100 18550597

[ref217] Nguyen D. K. Disteche C. M. (2006). Dosage compensation of the active X chromosome in mammals. Nat. Genet. 38, 47–53. doi: 10.1038/ng1705 16341221

[ref218] Nishizawa S. Benkelfat C. Young S. N. Leyton M. Mzengeza S. de Montigny C.. (1997). Differences between males and females in rates of serotonin synthesis in human brain. Proc. Natl. Acad. Sci. U. S. A. 94, 5308–5313. doi: 10.1073/pnas.94.10.5308 9144233 PMC24674

[ref219] Nøvik T. S. Hervas A. Ralston S. J. Dalsgaard S. Rodrigues Pereira R. Lorenzo M. J.. (2006). Influence of gender on attention-deficit/hyperactivity disorder in Europe – ADORE. Eur. Child Adolesc. Psychiatry 15, I15–I24. doi: 10.1007/s00787-006-1003-z, PMID: 17177011

[ref220] NSF. (2023). *Diversity and STEM: Women, Minorities, and Persons with Disabilities*. National Science Foundation 2023. Available at: https://ncses.nsf.gov/pubs/nsf23315/ (Accessed Sep 7, 2023).

[ref221] Nugent B. M. Wright C. L. Shetty A. C. Hodes G. E. Lenz K. M. Mahurkar A.. (2015). Brain feminization requires active repression of masculinization via DNA methylation. Nat. Neurosci. 18, 690–697. doi: 10.1038/nn.3988, PMID: 25821913 PMC4519828

[ref222] Ogawa S. Chester A. E. Hewitt S. C. Walker V. R. Gustafsson J. A. Smithies O.. (2000). Abolition of male sexual behaviors in mice lacking estrogen receptors alpha and beta (alpha beta ERKO). Proc. Natl. Acad. Sci. USA 97, 14737–14741. doi: 10.1073/pnas.250473597, PMID: 11114183 PMC18988

[ref223] Ogawa S. Eng V. Taylor J. Lubahn D. B. Korach K. S. Pfaff D. W. (1998). Roles of estrogen receptor-alpha gene expression in reproduction-related behaviors in female mice. Endocrinology 139, 5070–5081. doi: 10.1210/endo.139.12.6357, PMID: 9832446

[ref224] Okamoto M. Hojo Y. Inoue K. Matsui T. Kawato S. McEwen B. S.. (2012). Mild exercise increases dihydrotestosterone in hippocampus providing evidence for androgenic mediation of neurogenesis. Proc. Natl. Acad. Sci. USA 109, 13100–13105. doi: 10.1073/pnas.1210023109, PMID: 22807478 PMC3420174

[ref225] Olds T. Tomkinson G. Léger L. Cazorla G. (2006). Worldwide variation in the performance of children and adolescents: an analysis of 109 studies of the 20-m shuttle run test in 37 countries. J. Sports Sci. 24, 1025–1038. doi: 10.1080/02640410500432193, PMID: 17115514

[ref226] Ortiz J. Artigas F. Gelpí E. (1988). Serotonergic status in human blood. Life Sci. 43, 983–990. doi: 10.1016/0024-3205(88)90543-7 2459577

[ref227] Parsey R. V. Oquendo M. A. Simpson N. R. Ogden R. T. Van Heertum R. Arango V.. (2002). Effects of sex, age, and aggressive traits in man on brain serotonin 5-HT1A receptor binding potential measured by PET using [C-11]WAY-100635. Brain Res. 954, 173–182. doi: 10.1016/S0006-8993(02)03243-2, PMID: 12414100

[ref228] Paus T. (2010). Sex differences in the human brain: a developmental perspective. Prog. Brain Res. 186, 13–28. doi: 10.1016/B978-0-444-53630-3.00002-6 21094883

[ref229] Peters M. Lehmann W. Takahira S. Takeuchi Y. Jordan K. (2006). Mental rotation test performance in four cross-cultural samples (N = 3367): overall sex differences and the role of academic program in performance. Cortex 42, 1005–1014. doi: 10.1016/S0010-9452(08)70206-5, PMID: 17172180

[ref230] Pietschnig J. Penke L. Wicherts J. M. Zeiler M. Voracek M. (2015). Meta-analysis of associations between human brain volume and intelligence differences: how strong are they and what do they mean? Neurosci. Biobehav. Rev. 57, 411–432. doi: 10.1016/j.neubiorev.2015.09.017, PMID: 26449760

[ref231] Pike C. J. Nguyen T. V. V. Ramsden M. Yao M. Murphy M. P. Rosario E. R. (2008). Androgen cell signaling pathways involved in neuroprotective actions. Horm. Behav. 53, 693–705. doi: 10.1016/j.yhbeh.2007.11.006, PMID: 18222446 PMC2424283

[ref232] Pol H. E. H. Cohen-Kettenis P. T. Van Haren N. E. M. Peper J. S. Brans R. G. H. Cahn W.. (2006). Changing your sex changes your brain: influences of testosterone and estrogen on adult human brain structure. Eur. J. Endocrinol. 155, S107–S114. doi: 10.1530/eje.1.02248

[ref233] Proebstl L. Kamp F. Manz K. Krause D. Adorjan K. Pogarell O.. (2019). Effects of stimulant drug use on the dopaminergic system: a systematic review and meta-analysis of in vivo neuroimaging studies. Eur. Psychiatry 59, 15–24. doi: 10.1016/j.eurpsy.2019.03.003, PMID: 30981746

[ref234] Qin W. Liu C. Sodhi M. Lu H. (2016). Meta-analysis of sex differences in gene expression in schizophrenia. BMC Syst. Biol. 10:9. doi: 10.1186/s12918-015-0250-3 26818902 PMC4895727

[ref235] Ragland J. D. Coleman A. R. Gur R. C. Glahn D. C. Gur R. E. (2000). Sex differences in brain-behavior relationships between verbal episodic memory and resting regional cerebral blood flow. Neuropsychologia 38, 451–461. doi: 10.1016/S0028-3932(99)00086-X, PMID: 10683395 PMC4334366

[ref236] Raji C. A. Erickson K. I. Lopez O. L. Kuller L. H. Gach H. M. Thompson P. M.. (2014). Regular fish consumption and age-related brain gray matter loss. Am. J. Prev. Med. 47, 444–451. doi: 10.1016/j.amepre.2014.05.037, PMID: 25084680 PMC4171345

[ref237] Raji C. A. Merrill D. A. Eyre H. Mallam S. Torosyan N. Erickson K. I.. (2016). Longitudinal relationships between caloric expenditure and gray matter in the cardiovascular health study. J. Alzheimers Dis. 52, 719–729. doi: 10.3233/JAD-160057, PMID: 26967227 PMC4927887

[ref238] Rametti G. Carrillo B. Gómez-Gil E. Junque C. Segovia S. Gomez Á.. (2011b). White matter microstructure in female to male transsexuals before cross-sex hormonal treatment. A diffusion tensor imaging study. J. Psychiatr. Res. 45, 199–204. doi: 10.1016/j.jpsychires.2010.05.006, PMID: 20562024

[ref239] Rametti G. Carrillo B. Gómez-Gil E. Junque C. Zubiarre-Elorza L. Segovia S.. (2011a). The microstructure of white matter in male to female transsexuals before cross-sex hormonal treatment. A DTI study. J. Psychiatr. Res. 45, 949–954. doi: 10.1016/j.jpsychires.2010.11.007, PMID: 21195418

[ref240] Rametti G. Carrillo B. Gómez-Gil E. Junque C. Zubiaurre-Elorza L. Segovia S.. (2012). Effects of androgenization on the white matter microstructure of female-to-male transsexuals. A diffusion tensor imaging study. Psychoneuroendocrinology 37, 1261–1269. doi: 10.1016/j.psyneuen.2011.12.019, PMID: 22260939

[ref241] Reilly D. (2012). Exploring the science behind sex and gender differences in cognitive abilities. Sex Roles 67, 247–250. doi: 10.1007/s11199-012-0134-6

[ref242] Reiman E. M. Armstrong S. M. Matt K. S. Mattox J. H. (1996). The application of positron emission tomography to the study of the normal menstrual cycle. Hum. Reprod. 11, 2799–2805. doi: 10.1093/oxfordjournals.humrep.a019214, PMID: 9021395

[ref243] Reinius B. Saetre P. Leonard J. A. Blekhman R. Merino-Martinez R. Gilad Y.. (2008). An evolutionarily conserved sexual signature in the primate brain. PLoS Genet. 4:e1000100. doi: 10.1371/journal.pgen.1000100, PMID: 18566661 PMC2413013

[ref244] Riley J. L. Robinson M. E. Wise E. A. Myers C. D. Fillingim R. B. (1998). Sex differences in the perception of noxious experimental stimuli: a meta-analysis. Pain 74, 181–187. doi: 10.1016/S0304-3959(97)00199-1, PMID: 9520232

[ref245] Ritchie S. J. Cox S. R. Shen X. Lombardo M. V. Reus L. M. Alloza C.. (2018). Sex differences in the adult human brain: evidence from 5216 UK biobank participants. Cereb. Cortex 28, 2959–2975. doi: 10.1093/cercor/bhy109, PMID: 29771288 PMC6041980

[ref246] Robinson D. S. Sourkes T. L. Nies A. Harris L. S. Spector S. Bartlett D. L.. (1977). Monoamine metabolism in human brain. Arch. Gen. Psychiatry 34, 89–92. doi: 10.1001/archpsyc.1977.01770130091009 13761

[ref247] Rodriguez G. Warkentin S. Risberg J. Rosadini G. (1988). Sex differences in regional cerebral blood flow. J. Cereb. Blood Flow Metab. 8, 783–789. doi: 10.1038/jcbfm.1988.133 3192645

[ref248] Rossetti M. F. Cambiasso M. J. Holschbach M. A. Cabrera R. (2016). Oestrogens and Progestagens: synthesis and action in the brain. J. Neuroendocrinol. 28:402. doi: 10.1111/jne.12402 27306650

[ref249] Ruau D. Liu L. Y. Clark J. D. Angst M. S. Butte A. J. (2012). Sex differences in reported pain across 11,000 patients captured in electronic medical records. J. Pain 13, 228–234. doi: 10.1016/j.jpain.2011.11.002, PMID: 22245360 PMC3293998

[ref250] Ruigrok A. N. V. Salimi-Khorshidi G. Lai M. C. Baron-Cohen S. Lombardo M. V. Tait R. J.. (2014). A meta-analysis of sex differences in human brain structure. Neurosci. Biobehav. Rev. 39, 34–50. doi: 10.1016/j.neubiorev.2013.12.004, PMID: 24374381 PMC3969295

[ref251] Rutter M. Caspi A. Moffitt T. E. (2003). Using sex differences in psychopathology to study causal mechanisms: unifying issues and research strategies. J. Child Psychol. Psychiatry 44, 1092–1115. doi: 10.1111/1469-7610.00194, PMID: 14626453

[ref252] Sacher J. Neumann J. Okon-Singer H. Gotowiec S. Villringer A. (2013). Sexual dimorphism in the human brain: evidence from neuroimaging. Magn. Reson. Imaging 31, 366–375. doi: 10.1016/j.mri.2012.06.007 22921939

[ref253] Sadeghi M. Fakhrai A. (2000). Transsexualism in female monozygotic twins: a case report. Aust. N. Z. J. Psychiatry 34, 862–864. doi: 10.1080/j.1440-1614.2000.00804.x, PMID: 11037375

[ref254] Sakaki M. Mather M. (2012). How reward and emotional stimuli induce different reactions across the menstrual cycle. Soc. Pers. Psychol. Compass 6, 1–17. doi: 10.1111/j.1751-9004.2011.00415.x PMC3380631 22737180

[ref255] Sanacora G. Mason G. F. Rothman D. L. Behar K. L. Hyder F. Petroff O. A.. (1999). Reduced cortical γ-aminobutyric acid levels in depressed patients determined by proton magnetic resonance spectroscopy. Arch. Gen. Psychiatry 56, 1043–1047. doi: 10.1001/archpsyc.56.11.1043 10565505

[ref256] Santos E. M. Workman V. L. Paull G. C. Filby A. L. Van Look K. J. W. Kille P.. (2007). Molecular basis of sex and reproductive status in breeding zebrafish. Physiol. Genomics 30, 111–122. doi: 10.1152/physiolgenomics.00284.2006, PMID: 17374843

[ref257] Savic I. Arver S. (2011). Sex dimorphism of the brain in male-to-female transsexuals. Cereb. Cortex 21, 2525–2533. doi: 10.1093/cercor/bhr032, PMID: 21467211

[ref258] Schiller C. E. Meltzer-Brody S. Rubinow D. R. (2015). The role of reproductive hormones in postpartum depression. CNS Spectr. 20, 48–59. doi: 10.1017/S1092852914000480, PMID: 25263255 PMC4363269

[ref259] Schmitt D. P. Realo A. Voracek M. Allik J. (2008). Why can’t a man be more like a woman? Sex differences in big five personality traits across 55 cultures. J. Pers. Soc. Psychol. 94, 168–182. doi: 10.1037/0022-3514.94.1.168, PMID: 18179326

[ref260] Scholz B. Kultima K. Mattsson A. Axelsson J. Brunström B. Halldin K.. (2006). Sex-dependent gene expression in early brain development of chicken embryos. BMC Neurosci. 7:12. doi: 10.1186/1471-2202-7-12, PMID: 16480516 PMC1386693

[ref261] Schwarz J. M. Nugent B. M. McCarthy M. M. (2010). Developmental and hormone-induced epigenetic changes to estrogen and progesterone receptor genes in brain are dynamic across the life span. Endocrinology 151, 4871–4881. doi: 10.1210/en.2010-0142, PMID: 20702577 PMC2946142

[ref262] Seeman M. V. Lang M. (1990). The role of estrogens in schizophrenia gender differences. Schizophr. Bull. 16, 185–194. doi: 10.1093/schbul/16.2.185 2197713

[ref263] Segal N. L. (2006). Two monozygotic twin pairs discordant for female-to-male transsexualism. Arch. Sex. Behav. 35, 346–357. doi: 10.1007/s10508-006-9037-3, PMID: 16802182

[ref264] Shah N. M. Pisapia D. J. Maniatis S. Mendelsohn M. M. Nemes A. Axel R. (2004). Visualizing sexual dimorphism in the brain. Neuron 43, 313–319. doi: 10.1016/j.neuron.2004.07.008 15294140

[ref265] Shi L. Zhang Z. Su B. (2016). Sex biased gene expression profiling of human brains at major developmental stages. Sci. Rep. 6:21181. doi: 10.1038/srep21181, PMID: 26880485 PMC4754746

[ref266] Shors T. J. (2002). Opposite effects of stressful experience on memory formation in males versus females. Dialogues Clin. Neurosci. 4, 139–147. doi: 10.31887/DCNS.2002.4.2/tshors, PMID: 22033590 PMC3181678

[ref267] Simon L. R. Scott A. J. Figueroa Rios L. Zembles J. Masters K. S. (2023). Cellular-scale sex differences in extracellular matrix remodeling by valvular interstitial cells. Heart Vessel. 38, 122–130. doi: 10.1007/s00380-022-02164-2, PMID: 36070095 PMC10120251

[ref268] Simpson E. R. (2003). Sources of estrogen and their importance. J. Steroid Biochem. Mol. Biol. 86, 225–230. doi: 10.1016/S0960-0760(03)00360-1 14623515

[ref269] Singh M. Su C. (2013). Progesterone and neuroprotection. Horm. Behav. 63, 284–290. doi: 10.1016/j.yhbeh.2012.06.003, PMID: 22732134 PMC3467329

[ref270] Sisk C. L. Foster D. L. (2004). The neural basis of puberty and adolescence. Nat. Neurosci. 7, 1040–1047. doi: 10.1038/nn1326, PMID: 15452575

[ref271] Skuse D. H. (2005). X-linked genes and mental functioning. Hum. Mol. Genet. 14 Spec No 1, R27–R32. doi: 10.1093/hmg/ddi112, PMID: 15809269

[ref272] Slosman D. O. Chicherio C. Ludwig C. Genton L. de Ribaupierre S. Hans D.. (2001). (133)Xe SPECT cerebral blood flow study in a healthy population: determination of T-scores. J. Nucl. Med. 42, 864–870. PMID: 11390549

[ref273] Smith J. M. (1978). GRH volume 32 issue 3 Cover and Back matter. Genet. Res. 32, b1–b9. doi: 10.1017/S0016672300018693

[ref274] Smith S. S. Woolley C. S. (2004). Cellular and molecular effects of steroid hormones on CNS excitability. Cleve. Clin. J. Med. 71, S4–S10. doi: 10.3949/ccjm.71.Suppl_2.S4 15379294

[ref275] Sowell E. R. Peterson B. S. Kan E. Woods R. P. Yoshii J. Bansal R.. (2007). Sex differences in cortical thickness mapped in 176 healthy individuals between 7 and 87 years of age. Cereb. Cortex 17, 1550–1560. doi: 10.1093/cercor/bhl066, PMID: 16945978 PMC2329809

[ref276] Spear L. P. (2000). The adolescent brain and age-related behavioral manifestations. Neurosci. Biobehav. Rev. 24, 417–463. doi: 10.1016/S0149-7634(00)00014-2 10817843

[ref277] Squire L. R. (1992). Memory and the hippocampus: A synthesis from findings with rats, monkeys, and humans. Psychol. Rev. 99, 195–231. doi: 10.1037/0033-295x.99.2.195 1594723

[ref278] Staley J. K. Sanacora G. Tamagnan G. Maciejewski P. K. Malison R. T. Berman R. M.. (2006). Sex differences in diencephalon serotonin transporter availability in major depression. Biol Psychiatry 59, 40–47. doi: 10.1016/j.biopsych.2005.06.012 16139815

[ref279] Stanisić V. Lonard D. M. O’Malley B. W. (2010). Modulation of steroid hormone receptor activity. Prog. Brain Res. 181, 153–176. doi: 10.1016/S0079-6123(08)81009-6 20478437

[ref280] Stewart J. Rodaros D. (1999). The effects of gonadal hormones on the development and expression of the stimulant effects of morphine in male and female rats. Behav. Brain Res. 102, 89–98. 10403018 10.1016/s0166-4328(99)00002-9

[ref281] Stoet G. Geary D. C. (2013). Sex differences in mathematics and reading achievement are inversely related: within- and across-nation assessment of 10 years of PISA data. PLoS One 8:e57988. doi: 10.1371/journal.pone.0057988, PMID: 23516422 PMC3596327

[ref282] Stoet G. Geary D. C. (2018). The gender-equality paradox in science, technology, engineering, and mathematics education. Psychol. Sci. 29, 581–593. doi: 10.1177/0956797617741719, PMID: 29442575

[ref283] Su R. Rounds J. Armstrong P. I. (2009). Men and things, women and people: a meta-analysis of sex differences in interests. Psychol. Bull. 135, 859–884. doi: 10.1037/a0017364, PMID: 19883140

[ref284] Sullivan E. V. Rohlfing T. Pfefferbaum A. Full article: Longitudinal Study of Callosal Microstructure in the Normal Adult Aging Brain Using Quantitative DTI Fiber Tracking. Dev. Neuropsychol. 35, 233–256. doi: 10.1080/87565641003689556, PMID: 20446131 PMC2867078

[ref285] Swanson S. A. Crow S. J. Le Grange D. Swendsen J. Merikangas K. R. (2011). Prevalence and correlates of eating disorders in adolescents. Results from the national comorbidity survey replication adolescent supplement. Arch. Gen. Psychiatry 68, 714–723. doi: 10.1001/archgenpsychiatry.2011.22, PMID: 21383252 PMC5546800

[ref286] Tan A. Ma W. Vira A. Marwha D. Eliot L. (2016). The human hippocampus is not sexually-dimorphic: Meta-analysis of structural MRI volumes. NeuroImage 124, 350–366. doi: 10.1016/j.neuroimage.2015.08.050 26334947

[ref287] Tan M. Tan U. (2001). Sex difference in susceptibility to epileptic seizures in rats: importance of estrous cycle. Int. J. Neurosci. 108, 175–191. doi: 10.3109/00207450108986513, PMID: 11699190

[ref288] Tang Y. Hojatkashani C. Dinov I. D. Sun B. Fan L. Lin X.. (2010). The construction of a Chinese MRI brain atlas: a morphometric comparison study between Chinese and Caucasian cohorts. NeuroImage 51, 33–41. doi: 10.1016/j.neuroimage.2010.01.111, PMID: 20152910 PMC2862912

[ref289] Tapp H. S. Commane D. M. Bradburn D. M. Arasaradnam R. Mathers J. C. Johnson I. T.. (2013). Nutritional factors and gender influence age-related DNA methylation in the human rectal mucosa. Aging Cell 12, 148–155. doi: 10.1111/acel.12030, PMID: 23157586 PMC3572581

[ref290] Taylor B. J. Truman J. W. (1992). Commitment of abdominal neuroblasts in Drosophila to a male or female fate is dependent on genes of the sex-determining hierarchy. Development 114, 625–642. doi: 10.1242/dev.114.3.625, PMID: 1618132

[ref291] Tharpe N. (2011). Adverse drug reactions in women’s health care. J. Midwifery Womens Health 56, 205–213. doi: 10.1111/j.1542-2011.2010.00050.x 21535369

[ref292] Toffoletto S. Lanzenberger R. Gingnell M. Sundström-Poromaa I. Comasco E. (2014). Emotional and cognitive functional imaging of estrogen and progesterone effects in the female human brain: a systematic review. Psychoneuroendocrinology 50, 28–52. doi: 10.1016/j.psyneuen.2014.07.025, PMID: 25222701

[ref293] Trabzuni D. Ramasamy A. Imran S. Walker R. Smith C. Weale M. E.. (2013). Widespread sex differences in gene expression and splicing in the adult human brain. Nat. Commun. 4:2771. doi: 10.1038/ncomms3771, PMID: 24264146 PMC3868224

[ref294] Trachtenberg J. T. Chen B. E. Knott G. W. Feng G. Sanes J. R. Welker E.. (2002). Long-term in vivo imaging of experience-dependent synaptic plasticity in adult cortex. Nature 420, 788–794. doi: 10.1038/nature01273, PMID: 12490942

[ref295] Tukiainen T. Villani A. C. Yen A. Rivas M. A. Marshall J. L. Satija R.. (2017). Landscape of X chromosome inactivation across human tissues. Nature 550, 244–248. doi: 10.1038/nature24265, PMID: 29022598 PMC5685192

[ref296] United Nations: Office on Drugs and Crime. (2022). *Global study on homicide*. Available at: https://www.unodc.org/unodc/en/data-and-analysis/global-study-on-homicide.html (Accessed Jun 14, 2022).

[ref297] Van Wingen G. A. van Broekhoven F. Verkes R. J. Petersson K. M. Bäckström T. Buitelaar J. K.. (2008). Progesterone selectively increases amygdala reactivity in women. Mol. Psychiatry 13, 325–333. doi: 10.1038/sj.mp.4002030, PMID: 17579609

[ref298] Velasco E. R. Florido A. Milad M. R. Andero R. (2019). Sex differences in fear extinction. Neurosci. Biobehav. Rev. 103, 81–108. doi: 10.1016/j.neubiorev.2019.05.020, PMID: 31129235 PMC6692252

[ref299] Verplaetse T. L. Morris E. D. McKee S. A. Cosgrove K. P. (2018). Sex differences in the nicotinic acetylcholine and dopamine receptor systems underlying tobacco smoking addiction. Curr. Opin. Behav. Sci. 23, 196–202. doi: 10.1016/j.cobeha.2018.04.004, PMID: 31341936 PMC6656369

[ref300] Vigé A. Gallou-Kabani C. Junien C. (2008). Sexual dimorphism in non-Mendelian inheritance. Pediatr. Res. 63, 340–347. doi: 10.1203/PDR.0b013e318165b896, PMID: 18356736

[ref301] Wallen K. (2005). Hormonal influences on sexually differentiated behavior in nonhuman primates. Front. Neuroendocrinol. 26, 7–26. doi: 10.1016/j.yfrne.2005.02.001, PMID: 15862182

[ref302] Watkins K. E. Paus T. Lerch J. P. Zijdenbos A. Collins D. L. Neelin P.. (2001). Structural asymmetries in the human brain: a voxel-based statistical analysis of 142 MRI scans. Cereb. Cortex 11, 868–877. doi: 10.1093/cercor/11.9.868, PMID: 11532891

[ref303] Weber D. Skirbekk V. Freund I. Herlitz A. (2014). The changing face of cognitive gender differences in Europe. Proc. Natl. Acad. Sci. 111, 11673–11678. doi: 10.1073/pnas.1319538111, PMID: 25071201 PMC4136621

[ref304] Weiner M. de la Plata C. Julie Fields B. Womack K. Rosenberg R. Gong Y. H.. (2009). Brain MRI, Apoliprotein E genotype, and plasma homocysteine in American Indian Alzheimer disease patients and Indian controls. Curr. Alzheimer Res. 6, 52–58. doi: 10.2174/156720509787313952, PMID: 19199875 PMC2752625

[ref305] Weiss L. A. Abney M. Cook E. H. Ober C. (2005). Sex-specific genetic architecture of whole blood serotonin levels. Am. J. Hum. Genet. 76, 33–41. doi: 10.1086/426697, PMID: 15526234 PMC1196431

[ref306] Weiss E. M. Kemmler G. Deisenhammer E. A. Fleischhacker W. W. Delazer M. (2003). Sex differences in cognitive functions. Personal. Individ. Differ. 35, 863–875. doi: 10.1016/S0191-8869(02)00288-X

[ref307] Williams O. O. F. Coppolino M. George S. R. Perreault M. L. (2021). Sex differences in dopamine receptors and relevance to neuropsychiatric disorders. Brain Sci. 11:199. doi: 10.3390/brainsci11091199 34573220 PMC8469878

[ref308] Wise D. D. Felker A. Stahl S. M. (2008). Tailoring treatment of depression for women across the reproductive lifecycle: the importance of pregnancy, vasomotor symptoms, and other estrogen-related events in psychopharmacology. CNS Spectr. 13, 647–662. doi: 10.1017/S1092852900013742, PMID: 18704021

[ref309] Wissink E. M. Vihervaara A. Tippens N. D. Lis J. T. (2019). Nascent RNA analyses: tracking transcription and its regulation. Nat. Rev. Genet. 20, 705–723. doi: 10.1038/s41576-019-0159-6 31399713 PMC6858503

[ref310] Woolley C. S. (1998). Estrogen-mediated structural and functional synaptic plasticity in the female rat hippocampus. Horm. Behav. 34, 140–148. doi: 10.1006/hbeh.1998.1466, PMID: 9799624

[ref311] Xu J. Burgoyne P. S. Arnold A. P. (2002). Sex differences in sex chromosome gene expression in mouse brain. Hum. Mol. Genet. 11, 1409–1419. doi: 10.1093/hmg/11.12.1409 12023983

[ref903] Yang C. F. Chiang M. C. Gray D. C. Prabhakaran M. Alvarado M. Juntti S. A.. (2013). Sexually dimorphic neurons in the ventromedial hypothalamus govern mating in both sexes and aggression in males. Cell 153, 896–909. doi: 10.1016/j.cell.2013.04.017, PMID: 23663785 PMC3767768

[ref312] Yeung A. W. K. (2018). Sex differences in brain responses to food stimuli: a meta-analysis on neuroimaging studies. Obes. Rev. 19, 1110–1115. doi: 10.1111/obr.12697, PMID: 29806222

[ref313] Yoshii F. Barker W. W. Chang J. Y. Loewenstein D. Apicella A. Smith D.. (1988). Sensitivity of cerebral glucose metabolism to age, gender, brain volume, brain atrophy, and cerebrovascular risk factors. J. Cereb. Blood Flow Metab. 8, 654–661. doi: 10.1038/jcbfm.1988.112, PMID: 3417794

[ref314] Yu Y. Chen J. Li D. Wang L. Wang W. Liu H. (2016). Systematic analysis of adverse event reports for sex differences in adverse drug events. Sci Rep 6:24955. doi: 10.1038/srep24955 27102014 PMC4840306

[ref315] Zein J. G. Erzurum S. C. (2015). Asthma is Different in Women. Curr Allergy Asthma Rep 15:28. doi: 10.1007/s11882-015-0528-y, PMID: 26141573 PMC4572514

[ref316] Zell E. Krizan Z. Teeter S. R. (2015). Evaluating gender similarities and differences using metasynthesis. Am. Psychol. 70, 10–20. doi: 10.1037/a0038208, PMID: 25581005

[ref317] Zhang C. Cahill N. D. Arbabshirani M. R. White T. Baum S. A. Michael A. M. (2016). Sex and age effects of functional connectivity in early adulthood. Brain Connect. 6, 700–713. doi: 10.1089/brain.2016.0429, PMID: 27527561 PMC5105352

[ref318] Ziabreva I. Poeggel G. Schnabel R. Braun K. (2003). Separation-induced receptor changes in the hippocampus and amygdala of *Octodon degus*: influence of maternal vocalizations. J. Neurosci. 23, 5329–5336. doi: 10.1523/JNEUROSCI.23-12-05329.2003, PMID: 12832558 PMC6741186

[ref319] Zubiaurre-Elorza L. Junque C. Gómez-Gil E. Guillamon A. (2014). Effects of cross-sex hormone treatment on cortical thickness in transsexual individuals. J. Sex. Med. 11, 1248–1261. doi: 10.1111/jsm.12491, PMID: 24617977

[ref320] Zubieta J. K. Dannals R. F. Frost J. J. (1999). Gender and age influences on human brain mu-opioid receptor binding measured by PET. Am. J. Psychiatry 156, 842–848. doi: 10.1176/ajp.156.6.842, PMID: 10360121

